# The disconnect between researcher ambitions and reality in achieving impact in the Earth & Environmental Sciences – narrowing the gap

**DOI:** 10.12688/f1000research.28324.1

**Published:** 2021-01-19

**Authors:** Andrew Kelly, Victoria Gardner, Anna Gilbert

**Affiliations:** 1 Taylor & Francis Group, Abingdon, OX14 4RN, UK

**Keywords:** academic publishing, Earth Sciences, Environmental Sciences, research journals, research assessment, survey, Sustainable Development Goals, SDGs

## Abstract

**Background:** There is an increasing desire for research to provide solutions to the grand challenges facing our global society, such as those expressed in the UN SDGs (“real-world impact”). Herein, we consider whether the frameworks that underpin the research endeavour are appropriately oriented to support these aspirations and maximize the capability of research to achieve these goals.

**Methods:** We conducted a survey of authors who had published in >100 of our Earth & Environmental Science journals. The survey was sent to just under 60,000 authors and we received 2,695 responses (4% response rate).

**Results:** Respondents indicated that the majority of their research in the Earth & Environmental Sciences is currently concerned with addressing urgent global needs or that this will become a priority in the future; however, the impetus seems to be altruistic researcher desire, rather than incentives or support from publishers, funders, or their institutions. Indeed, when contextualised within other forms of impact, respondents indicated that citations or downloads were more important to them than contributing to tackling real-world problems. Herein, we analyse survey feedback, suggest the presence of a misalignment between researcher ambition and current realities, and discuss the role and value of the research journal in forming new connections for their researchers, both within and without academia.

**Conclusions:** At present, it seems that this laudable ambition of achieving real-world impact is seemingly being lost amidst the realities of being a researcher. We offer for comment a series of suggestions, with the aim of simulating discussion and collective action to tackle these challenges as a community.

## Introduction

Although one of the fundamental tenets of the research endeavour is about exploration and curiosity, calls to increase R&D spending by nations have been allied with an expectation for research to have “impact” ^1^. Impact has many forms – some are easily quantifiable, for example every dollar invested in the Human Genome Project returned $141 to the US economy ^2^, whereas others are harder to quantify and often tie into complex interdisciplinary issues, which require long-term commitment and investment – the UN’s Sustainable Development Goals (SDGs) and the missions of Horizon Europe serving as prime examples ^3, 4^. This drive for research to solve ‘the Grand Challenges of our time’ ^5^ has acquired increased urgency during the Covid-19 pandemic, but has long been discussed in the context of global problems, including climate change, food security, and an aging global population. Where once there was a comment that we no longer needed experts ^6^, trust in research and researchers to provide solutions to real-world problems is currently high, with seven in ten people commenting before the Covid-19 pandemic that science benefits them ^7^, and trust in advice from ‘qualified scientists and researchers’ growing even faster in the face of the pandemic ^8, 9^. 

How do these aspirations manifest at the level of those carrying out research? Primarily, it’s through research-assessment mechanisms linked to institutional or grant funding, with citations being the primary currency of success or progress. The UK’s Research Excellence Framework (REF) ^10^ exercise has previously linked to the Pathways to Impact initiative ^11^, with contributions to the REF expected to provide impact case studies alongside their submissions that showcase their social or economic impact. It can be challenging to see how answering one research question can create a chain reaction that resonates at a much-higher level, still less how interrelated research questions contribute to solutions to these complex and interdisciplinary issues.

The role that academic publishing plays in the advancement of research is often understood to comprise validation (through the peer-review process), publication (participation in the scholarly record), curation (preservation of the work to ensure its availability in perpetuity), and dissemination (to relevant communities). However, it is becoming increasingly apparent that the value of a research journal is much broader than this, additionally fostering collaboration, network-building (both within core and adjacent fields), and career development ^12^. 

Therefore, it is essential that the mechanisms and drivers that collectively influence where an author chooses to publish their research support their ability to publish in the journals that are most relevant to their work; that is, where their research is most likely to be found, read, cited, and iterated upon by those working in the same and adjacent disciplines, as well as by those working in policy-making, lobbying, or advisory capacities. However, such drivers and pressures, both personal and external, are varied and nuanced, as are our authors’ expectations for what impact that their work might have once it has been published.

It is in this context that we undertook the *2020 Impact Assessment of Earth & Environmental Sciences Research: Author Survey*. The survey was designed to achieve three main aims. To understand:

- what drives our communities to choose the topics that they research;
- what drives our communities to choose the journals that they publish in; and
- what type(s) of impact they are most looking for from their work.

We investigated what benefits publishing in our journals could impart on both the research and on the authors following publication, and we looked at to what extent global challenges, such as those expressed by the UN SDGs, were shaping researcher ambitions. This report describes the results that we obtained and considers how the frameworks that are currently in place in academic publishing, such as those around securing research funding, researcher assessment, and career progression, are shaping the desires and decisions of our researchers, either in support of, or in opposition to, these desires.

## Methods

In Spring 2020, Taylor & Francis surveyed authors from across our Earth & Environmental Sciences portfolio. The survey (see *Extended data* ^13^), hosted on Alchemer (formerly SurveyGizmo), was emailed to authors using Salesforce Marketing Cloud. It was sent to just under 60,000 authors and received 2,695 responses (4% response rate).

The survey comprised 23 questions: section A (Q1 & 2) = multiple choice questions to clarify the article that the survey responses related to; section B (3 & 4) = multiple choice questions with the option of prose responses relating to the choice of journal; section C (5–10) = multiple choice questions with the option of prose responses relating to the downstream value of publishing the article for both the work and the author; section D (11–20) = largely multiple choice with the option of prose relating to the impact of the work, the motivation for undertaking the work, and the ability of the work to tackle real-world problems and influence policy change. Questions 13, 15, 18, and 20 were solely prose responses. Section E (21–23) = demographic questions.

A confidentiality and privacy statement was provided on the first page of the survey, which outlined how the data would be used. Consent to participate in the survey was implied by the authors who clicked through to complete the questionnaire after reading this statement and the instructions given in the invitation email. The data are fully anonymized and no sensitive personal data regarding the respondents were collected. To protect the anonymity of the respondents, all prose responses to the free-text questions (questions 13, 15, 18, and 20) have been omitted from the shared dataset. Written informed consent was not sought due to the low-risk nature of the research.

The survey responses include authors from 102 journals in the Earth & Environmental Sciences portfolio, and the geographical distribution of responses was similar to that of authors in the portfolio. Therefore, we can be reasonably confident that our responses are representative of Taylor & Francis authors in our Earth & Environmental Sciences journals.

### Data analysis

Confidence intervals have been calculated for certain parts of our analysis where we are comparing groups of different sizes within the survey, and we are only reporting on differences that are statistically significant. Microsoft Excel was used to prepare the tables and charts. Confidence intervals were calculator by using the Creative Research Systems sample size calculator ^14^.

***A note about error bars and statistical significance.*** The country-comparison charts presented in this report include error bars, which plot the confidence intervals for the percentages shown. When making comparisons, error bars are useful as a visual means of demonstrating the range that likely contains the true overall value for each country in the chart. If the error bars for two or more countries overlap, we should be cautious about making substantive conclusions about any differences, because they may not be statistically significant. Therefore, only clearly statistically significant differences are included in the comparisons presented herein.

## Results

### SDG-relevance of earth and environment research – the current picture

***Why do researchers undertake the research that they do?*** It is a fundamental question and the answer is multifaceted, varying by career stage, geography, and subject discipline. However, the publication of the SDGs by the UN in September 2015, which had the stated aim of providing a “a shared blueprint for peace and prosperity for people and the planet, now and into the future” ^3^, allows us an opportunity to frame the question in such a way that gets to the core of what researchers hope to achieve through their work, that is: “ *do researchers study topics that contribute, either directly or indirectly, to the tackling of real-world problems?*”.

**Figure 1.**
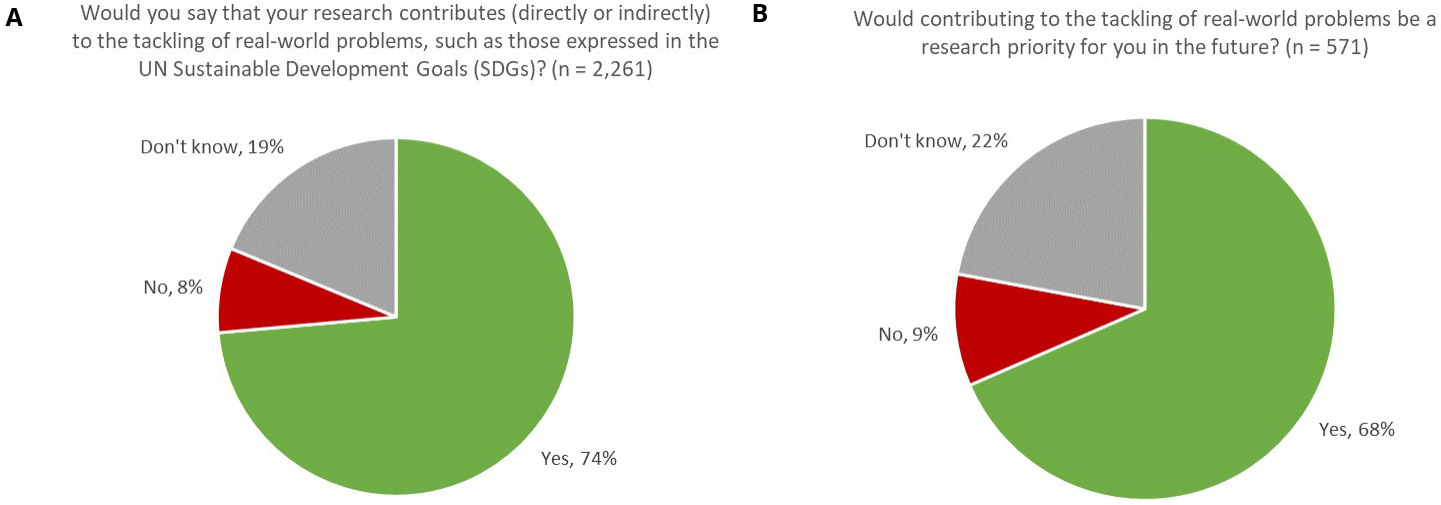
Proportion of respondents whose research contributes to tackling real-world problems, now ( **A**) and in the future ( **B**).

***The contribution of research.*** Comprising such urgent needs as Clean Water and Sanitation (SDG 6) and Climate Change (SDG 13) and tackling threats to Life on Land (SDG 15) and Life below Water (SDG 14), one might readily anticipate that a high proportion of research in the Earth and Environmental Sciences would have a part to play in meeting the needs expressed by the SDGs. Indeed, 74% of respondents indicated that their research contributed (directly or indirectly) to the tackling of real-world problems, such as those expressed by the UN SDGs ( Figure 1). Furthermore, overall, 90% of respondents indicated that their work either currently contributed to meeting real-world problems or that it would be a priority for them in the future. Therefore, we might infer that, at least in the Earth & Environmental Sciences, it is a strong research imperative for our authors that their work contributes to the tacking of real-world problems.

Such a high percentage aligns with the voices of our journal editors, who, in contributing to our recent publication “Sustainable Development Goals in the Earth and Environmental Sciences” ^15^, expounded the variety, breadth, and richness of research that their journals and subject areas have to offer in tackling the challenges laid out in the SDGs.

In our survey, whilst younger researchers were slightly more likely to undertake this type of research (76% of respondents aged under 50 answered “Yes” compared with 70% of respondents aged 50 or older, with resolved confidence intervals), the difference was not very pronounced, thus suggesting that this is a multi-generational aspiration, rather than one solely driven by early-career researchers.

### Why is addressing real-world challenges a research priority for our authors?

**Figure 2.**
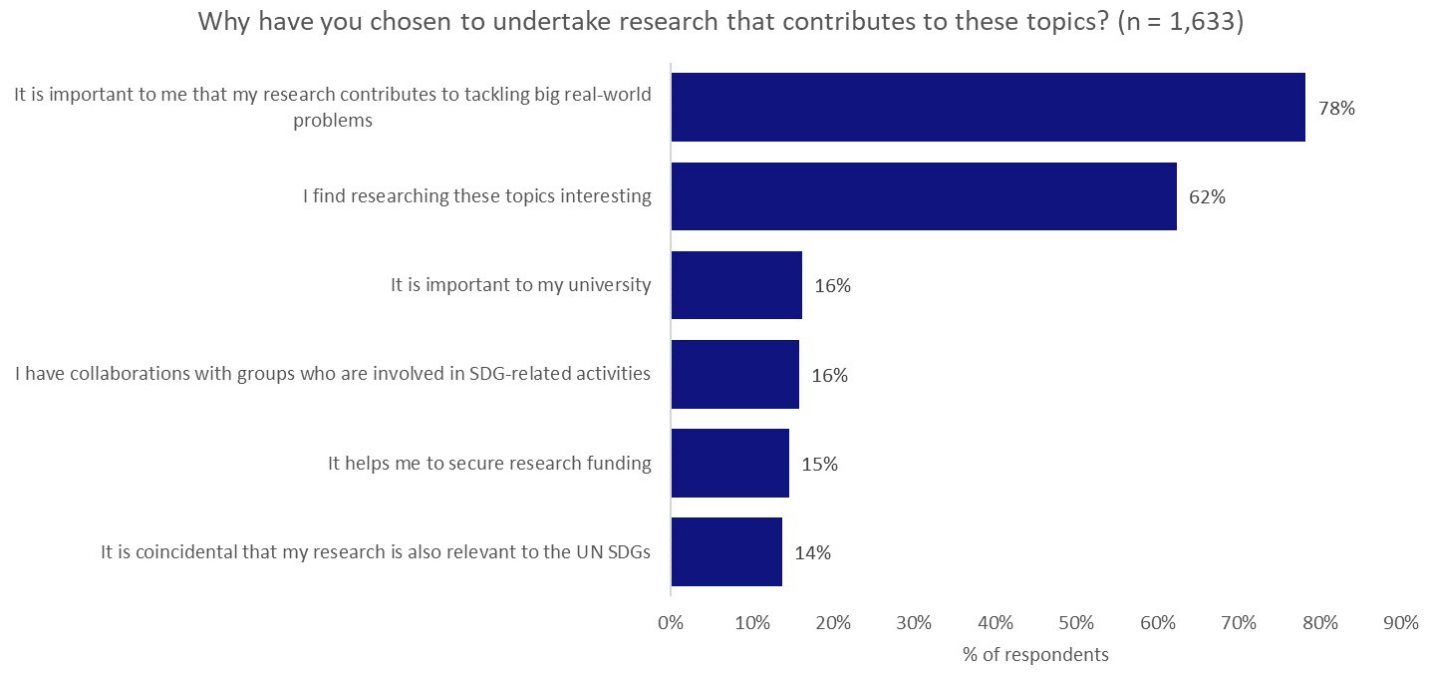
Motivating factors for investigating topics with applications to real-world problems.

To understand a bit more about the motivating factors that sit behind the decision of our researchers to investigate topics that have application to real-world problems, we asked “Why have you chosen to undertake research that contributes to these topics?” ( Figure 2). The responses to this question presented a clear split between internal drivers—personal interest (62%) and the desire to contribute to addressing real-world problems (78%)—and external drivers, such as encouragement from a university, other collaborators, or improved opportunities to secure research funding, with internal drivers and aspirations being the greater motivators.

We find it surprising that the influence of funders (15%) and institutions (16%) was only narrowly more influential than coincidence (14%) in prompting research that is skewed towards meeting these global challenges.

### Ambitions vs reality

**Figure 3.**
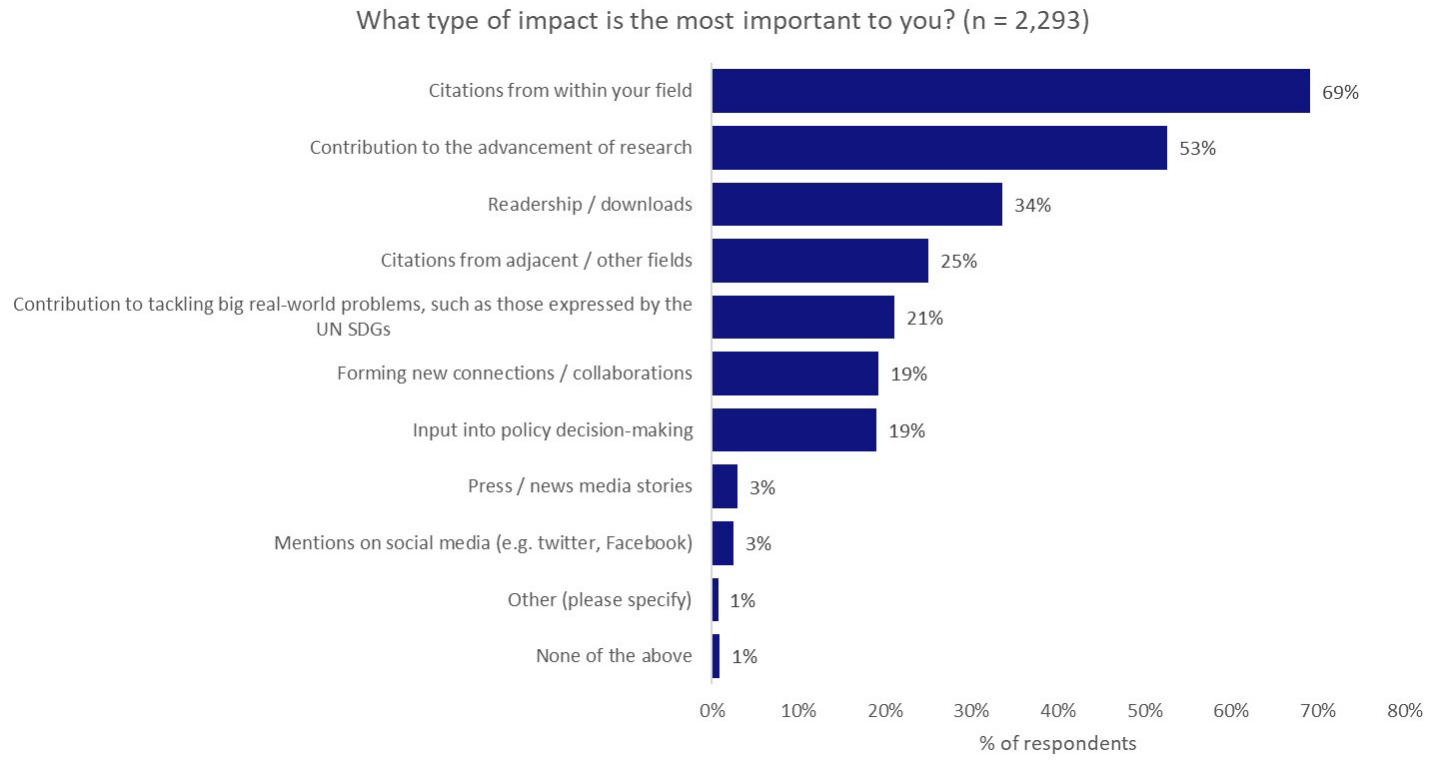
Most-preferred type of impact (maximum of three selections).

We saw the greatest gap between aspiration and reality when we asked what type of impact was most important to respondents— with a maximum of three selections. The most-preferred type of impact was citations from within the same field (69%), over against contribution to the advancement of research (53%), contribution to tackling ‘real-world’ problems, such as those expressed by the UN SDGs (21%), and input into policy decision-making (19%; Figure 3).

Interestingly, having seen the strong desire of authors to undertake research with real-world application in the earlier questions, when compared with other types of impact, contributing to the tacking of real-world problems dropped to fifth in the list (21%), behind citations from within the same field (69%) and from adjacent/other fields (25%), and achieving a large readership (34%).

We note that some respondents may have felt that citations were a necessary step in contributing to the advancement of research or in tackling real-world problems, through knowledge sharing and discussion, as the reasons for these selections weren’t probed further. However, as the question asked what the most important type of impact was to the researcher (“ *to you*”), we think this is unlikely to be a significant line of thought.

Input into policy decision-making, “where the rubber meets the road” for much of the national-scale change that is required to meet the needs captured by the UN SDGs (19%), placed further down the list, on par with forming new collaborations (19%). Only attention from the press (3%) and attention on social media (3%) ranked lower.

### The role of the journal

**Figure 4.**
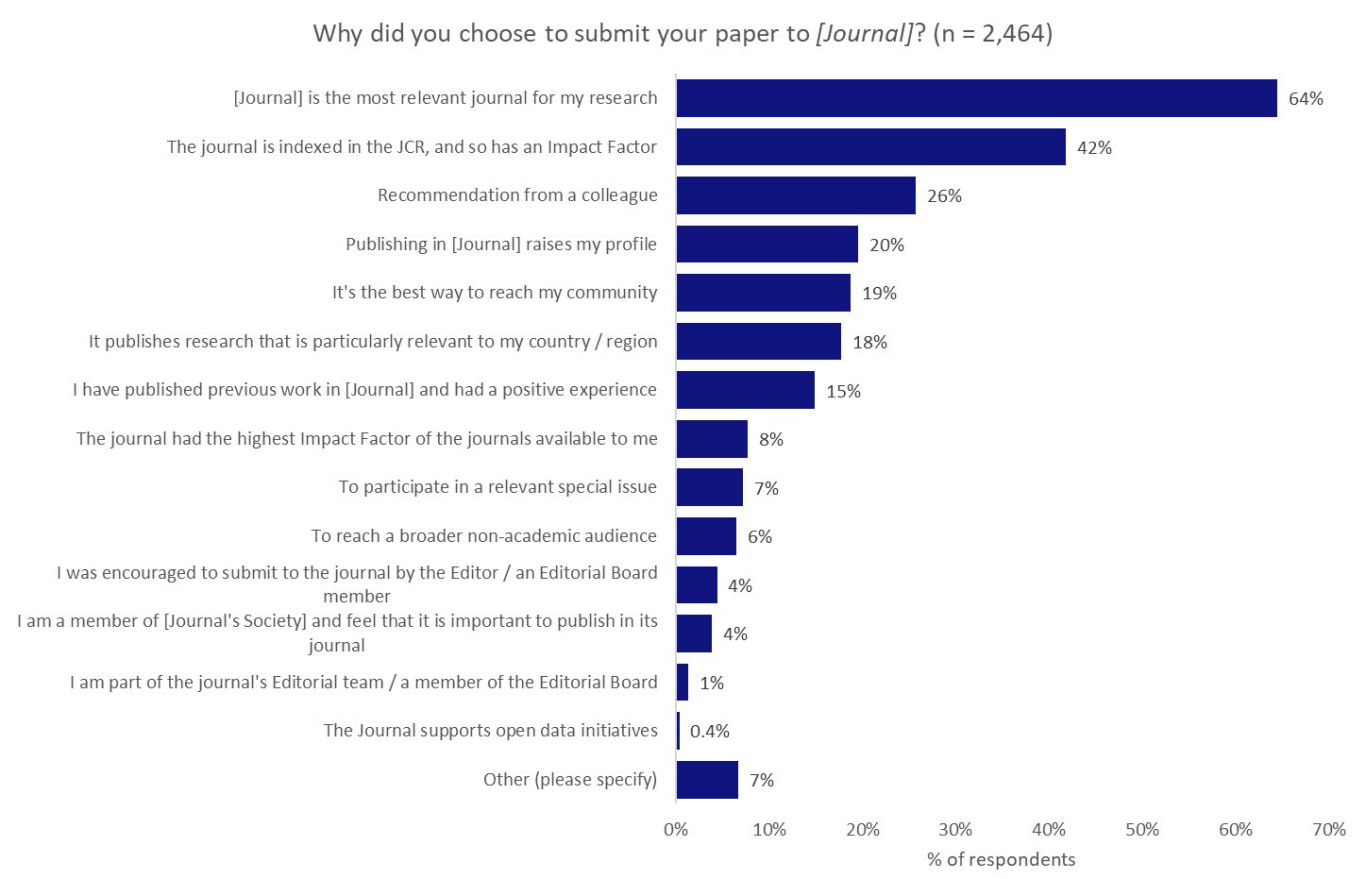
Motivating factors for the choice of journal.

Feedback from respondents has indicated two key points: the aspiration of researchers to contribute to the tackling of real-world problems with their work, compared with a focus on citations as the key measure of impact. What role might the choice of a journal have to play in serving either of these aims? To investigate this further, we asked all of the respondents why they submitted their paper to the journal their work was published in, and also whether this journal was their first choice ( Figure 4).

The predominant factor in determining the selection of a journal was its relevance to the author’s research, and 64% of respondents indicated this was one of the most-influential factors underpinning their choice of journal. Interesting, reaching a broader non-academic audience (which we might link to the desire to contribute to resolving real-world challenges) came quite far down the list, with only 6% of respondents noting that this was an influential factor in their choice of journal.

On asking our authors whether the journal that they published in was their first choice, 75% indicated that it was, whilst 19% indicated that it was their second choice and 6% indicated that they had submitted their paper to more than one other journal before publishing it.

In this context, it is perhaps unsurprising to see that a journal having an Impact Factor was important to 42% of respondents. Interestingly, although 42% of authors indicated that the journal having an Impact Factor was an important factor in their decision-making, only 8% indicated that they chose the journal because it had the highest available Impact Factor, thus indicating that the presence of an Impact Factor was more important than the score itself. Many institutions, policymakers, and funders are keen to reduce emphasis on the Impact Factor as part of research assessment practices ^16^, so there is perhaps a misalignment in the priorities of researchers, as opposed to their institutions and funders.

### What wider value can research journals offer to our academic communities?

To investigate if (and if so, how effectively) publishers support the essential profile-raising and network-building needs of our research communities, we asked survey respondents about the opportunities for network-building and recognition that publishing in the journal had afforded them since they published their work.

**Figure 5.**
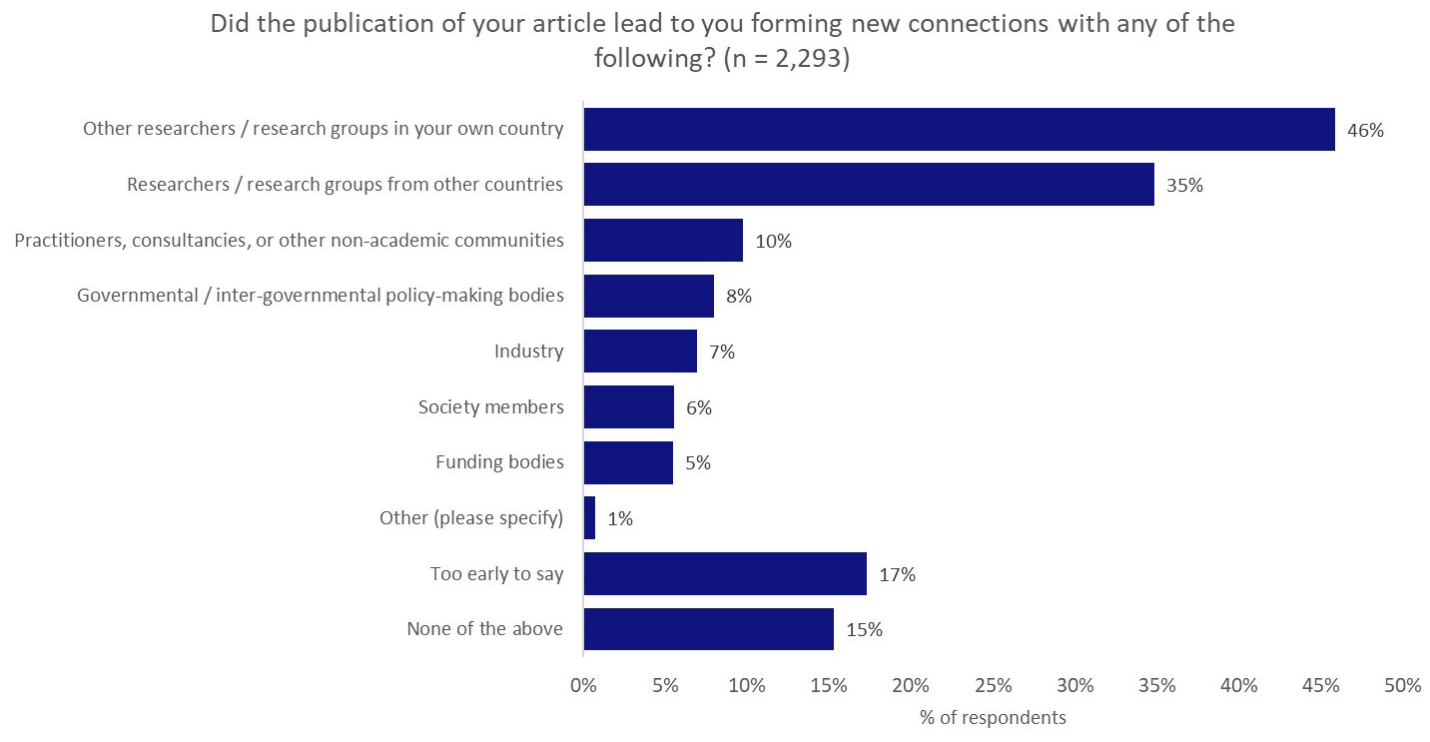
Influence of publishing in a journal on the formation of new academic and non-academic connections.

When asked if the publication of their article led to the formation of new connections with a range of different groups, almost half of respondents (46%) indicated that the publication of their article led to their forming new connections with researchers/research groups in their own country, whilst 35% formed new connections with researchers/research groups from other countries ( Figure 5). From this feedback, we infer that publications and journals have a vital and valuable role in facilitating global knowledge sharing within subject communities.

Survey feedback highlighted the positive influence that journals and publishers can have in forming new academic collaborations. It is important to note, however, that this “matchmaking” effect does not appear to be as profound in non-academic contexts, with only 7% of respondents noting that they were approached about non-academic collaborations after the publication of their work (however, 48% of these respondents did note that their work influenced or greatly influenced this approach). Furthermore, only 10% of respondents formed new connections with practitioners, consultancies, or other non-academic communities as a result of publishing their work; only 8% formed new connections with governmental/inter-governmental policy-making bodies; and fewer still formed new connections with industry (7%) or funding bodies (5%).

**Figure 6.**
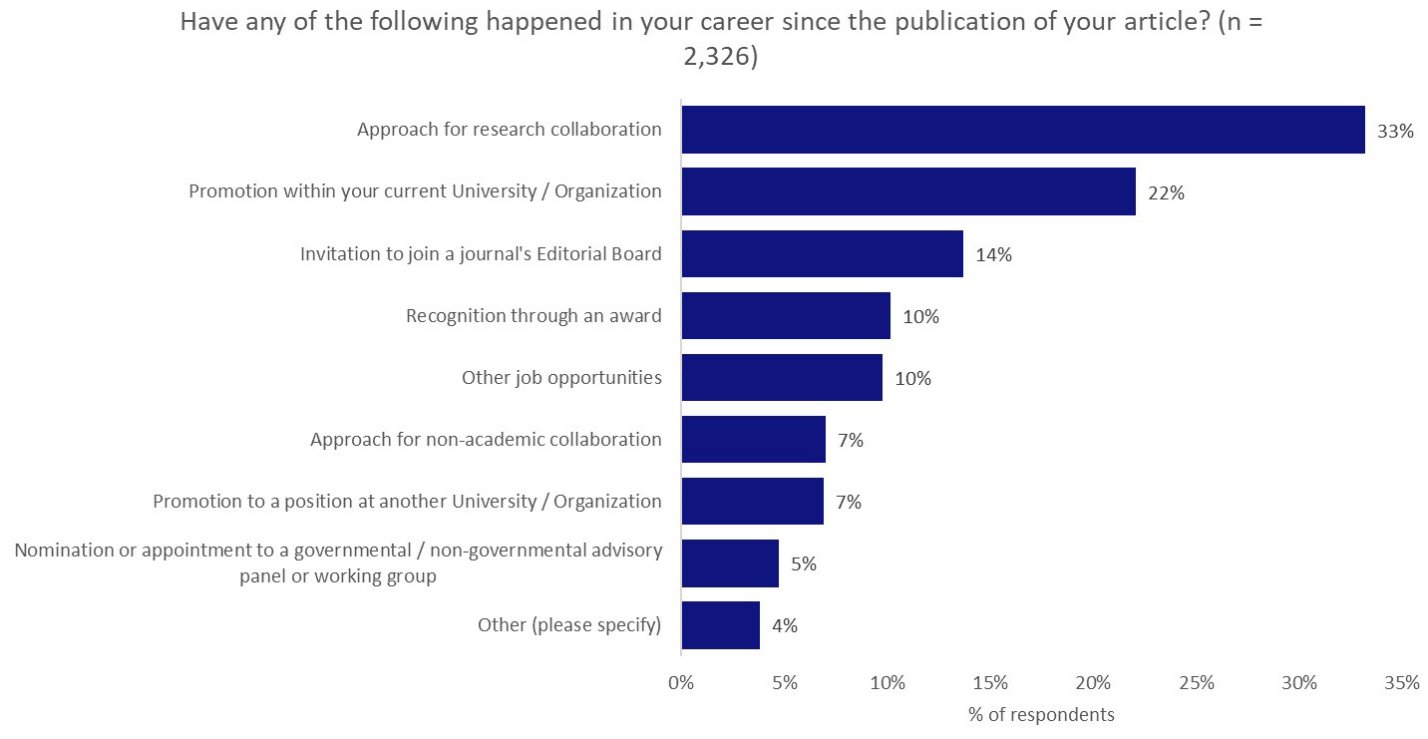
Proportion of respondents who achieved the noted career developments since the publication of their article.

**Figure 7.**
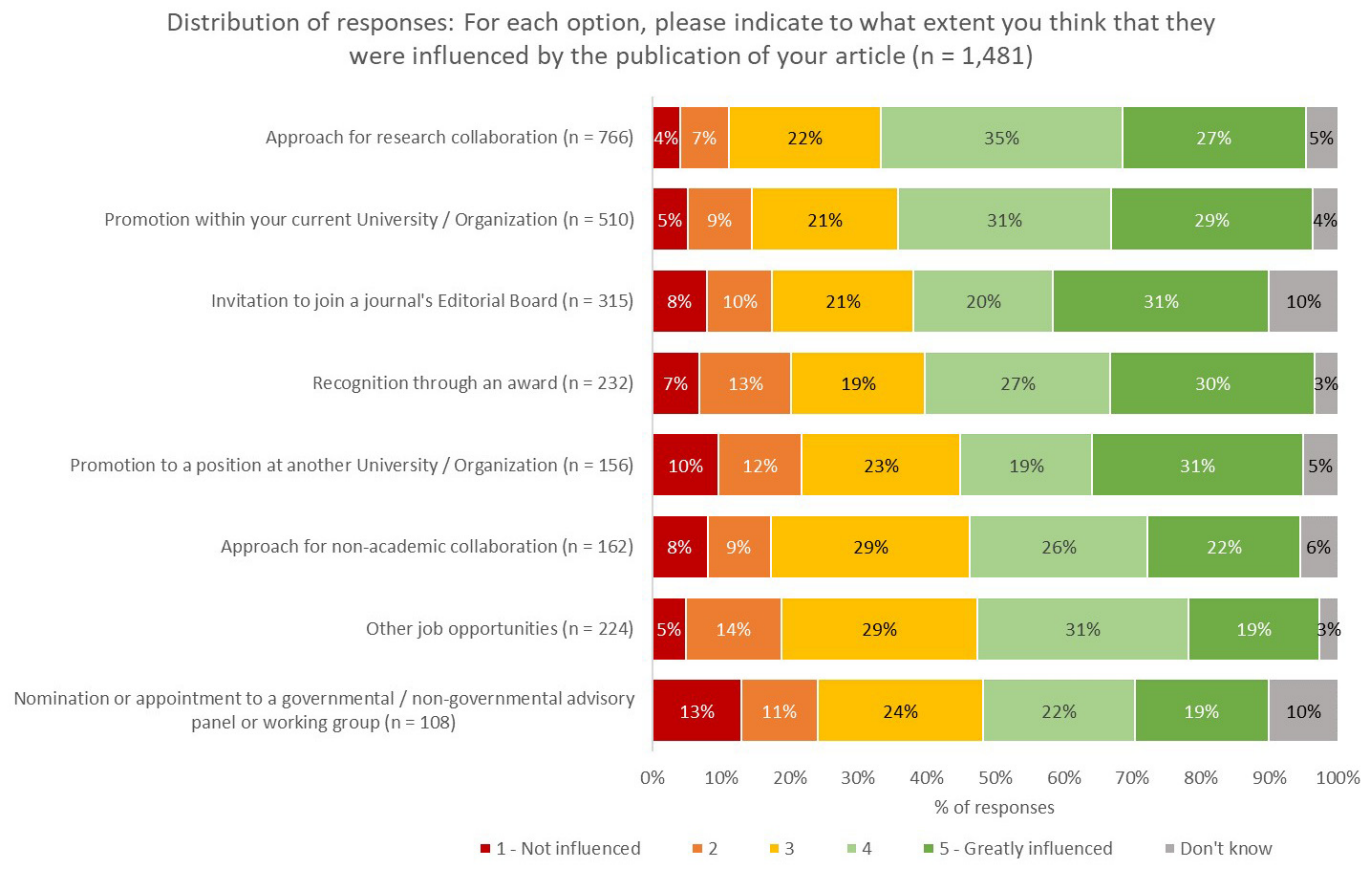
Influence of the journal publication on these career developments.

As shown in Figure 6, one third of respondents (33%) indicated that, since the publication of their article, they had been approached regarding a potential research collaboration, with two thirds of these respondents noting that publication of their work influenced or greatly influenced this approach ( Figure 7). In this regard, publishers and journals play a vital matchmaking role in linking together researchers for further collaboration.

### Comparing priorities across different geographies

Based on survey feedback, authors based in different regions appear to place different emphases on the criteria that shape their choice of journal, and their views around impact.

**Figure 8.**
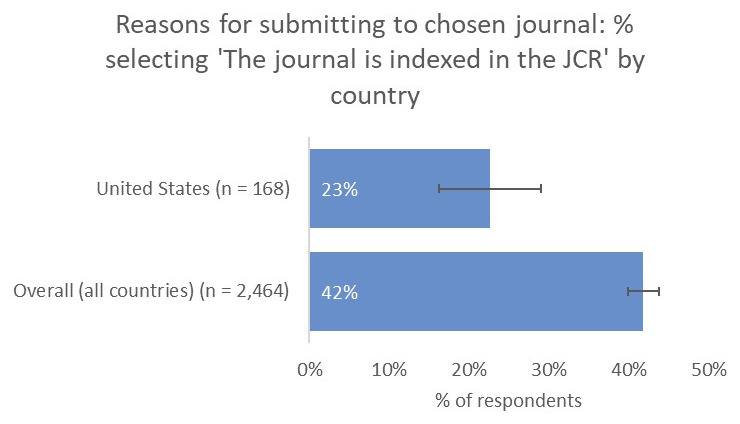
Relative importance of indexing in the JCR in determining choice of journal for US-based authors.

***United States.*** Fewer respondents based in the United States indicated that having an Impact Factor was an important criterion in determining their choice of journal compared to the overall average (23% vs 42%). This same lower emphasis on the Impact Factor is seen in our post-publication author survey, which is sent to authors in all subject areas and all geographies, with respondents from the US rating this as a less-important factor in determining their choice of journal compared with the global average ( Figure 8) ^17^.

**Figure 9.**
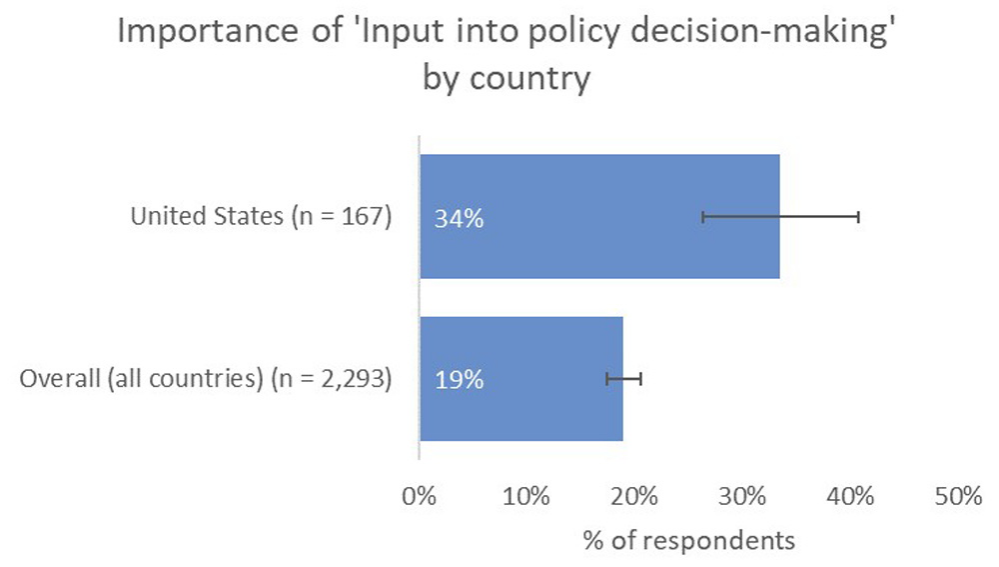
Relative importance of input into decision making for US-based authors.

Instead, US-based authors placed more value on real-world types of impact than the global average, with a higher proportion indicating that contribution to tackling big real-world problems, such as those expressed by the UN SDGs, was one of the most-important types of impact to them (29%), and a much-higher proportion indicating that having an input into policy decision-making was important to them (34% vs 19% overall; Figure 9).

**Figure 10.**
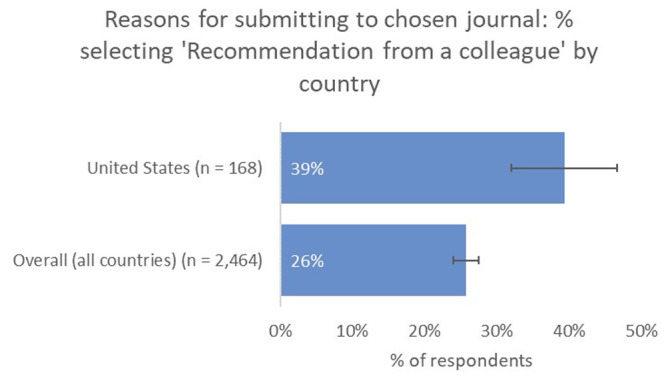
Relative importance of recommendation from a colleague in determining choice of journal for US-based authors.

Recommendation from a colleague (39% vs 26% overall) and the journal’s capacity to reach a broader non-academic audience (13% vs 7% overall) were also deemed to be much more important factors as a means of identifying a suitable journal for US-based respondents, compared to the global average ( Figure 10).

***China.*** Responses from researchers based in China largely reflected the overall results, both in the most-important types of impact to them and the most-important factors that influence their choice of journal. As in other territories, respondents from China indicated that receiving citations from within the same field was one of the most-important types of impact for them (72%), followed by contribution to the advancement of research (49%) and readership/downloads (33%).

**Figure 11.**
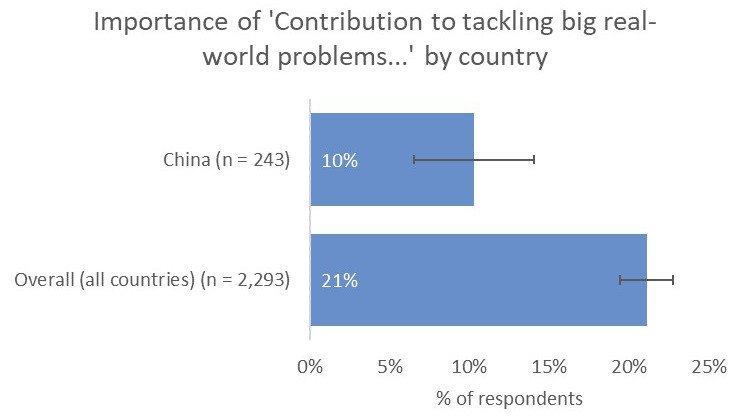
Relative importance of tackling real-world problems to China-based authors.

**Figure 12.**
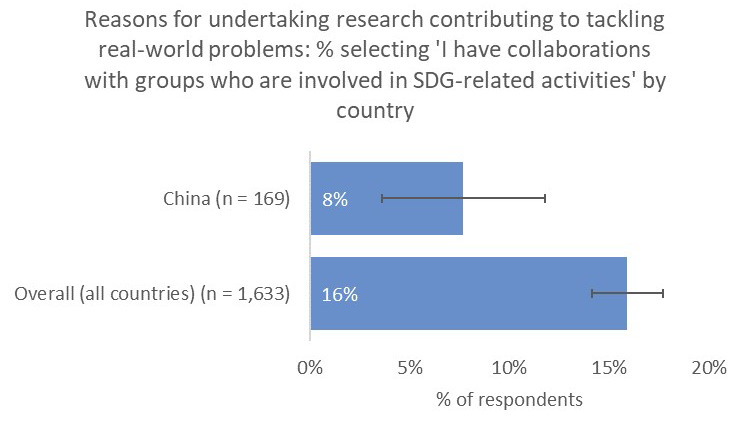
Relative proportion of researchers with collaborators who are involved in SDG-related activities.

However, one noticeable distinction was the relative unimportance of having a real-world impact in terms of contribution to tackling real-world problems (10% vs 21% overall; Figure 11) and input into policy decision making (8% vs 19% overall), perhaps because China-based respondents were less likely to have collaborations with groups who were involved in SDG-related activities (8% vs 16% overall; Figure 12).

**Figure 13.**
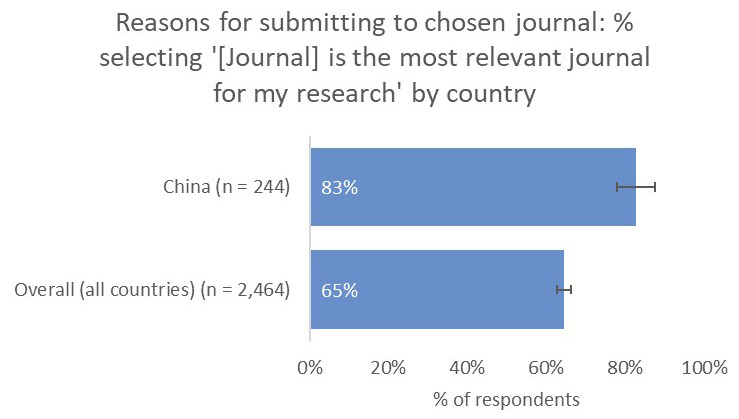
Relative importance of a journal’s relevance in determining choice of journal for China-based authors.

Conversely, for China-based researchers, the relevance of a journal to their work was a much-more-important consideration (83%) compared to the global average (65%) and compared to authors based in the US (59%) and Europe (49%), whilst whether the journal had an Impact Factor was as important to Chinese respondents (44%) as the overall average (42%; Figure 13).

***UK and Europe.*** Respondents from the UK and Europe closely followed the global averages for both the types of impact that were most important and the most-important factors in determining the choice of journal.

**Figure 14.**
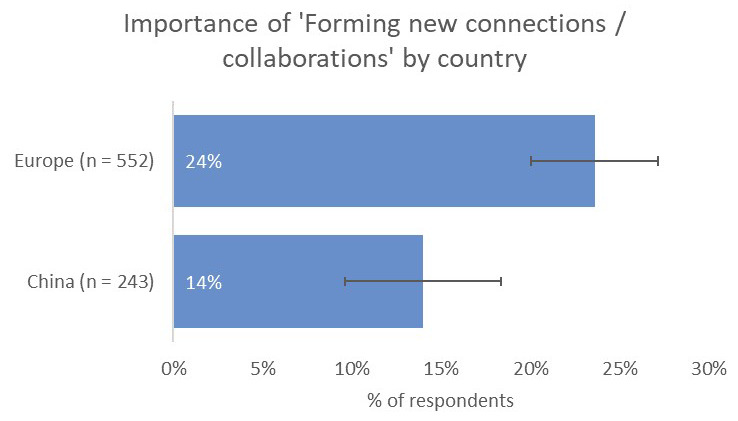
Relative importance of new collaborations as a form of impact for Europe- and China-based authors.

Respondents from the UK and Europe indicated that receiving citations from within the same field was the most-important type of impact to them (73%), followed by contribution to the advancement of research (50%) and readership/downloads (33%). Where respondents based in the UK and Europe differed was in the prospect of forming new collaborations (24%), which they considered to be a more-important type of impact than for respondents from India (16%) and China (14%; Figure 14).

**Figure 15.**
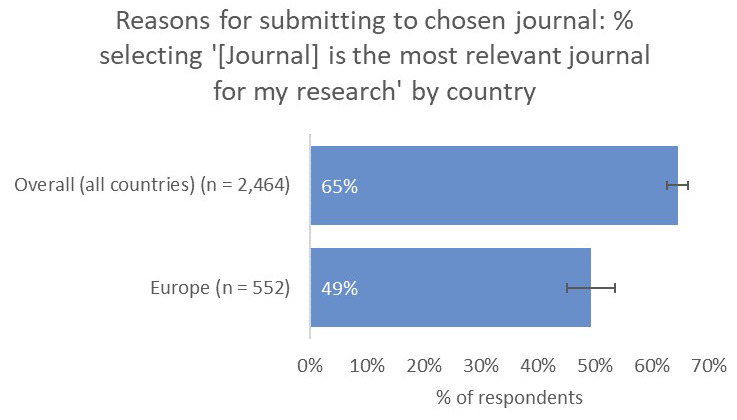
Relative importance of relevance in determining choice of journal for UK & Europe-based authors.

Interestingly, and perhaps related to the premium placed on network-building outside of their own subject communities, only 49% of respondents from the UK and Europe said that their work was published in the most-relevant journal, much lower than all other territories (65% average; Figure 15).

***India.*** Respondents from India again closely followed the global averages in terms of the types of impact that were most important and the most-important factors in determining the choice of journal. However, there were two distinct points of divergence.

**Figure 16.**
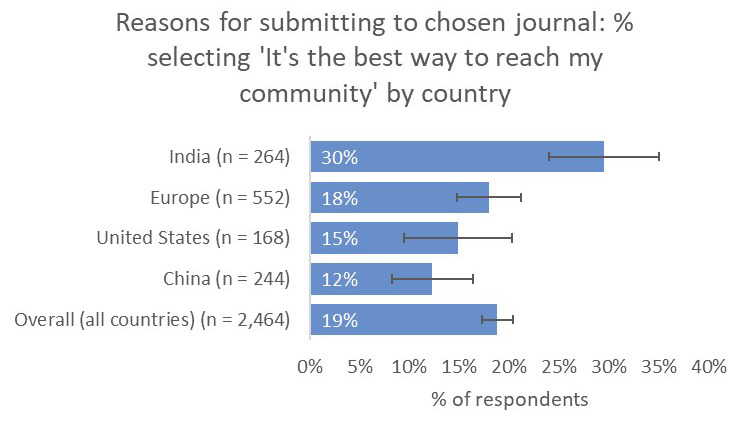
Relative importance of reaching a target community between the demographics considered.

Compared to the global average, respondents from India placed significantly greater importance on the relevance of the journal for their work (87% vs 65% overall), comparable to respondents from China (83%) and significantly higher than respondents from the US (59%) and the UK and Europe (49%). Similarly, respondents from India placed much greater importance on the journal’s capability to reach their community (30%) compared to respondents from China (12%), the US (15%), and UK and Europe (18%), as well as to the overall average (19%; Figure 16).

**Figure 17.**
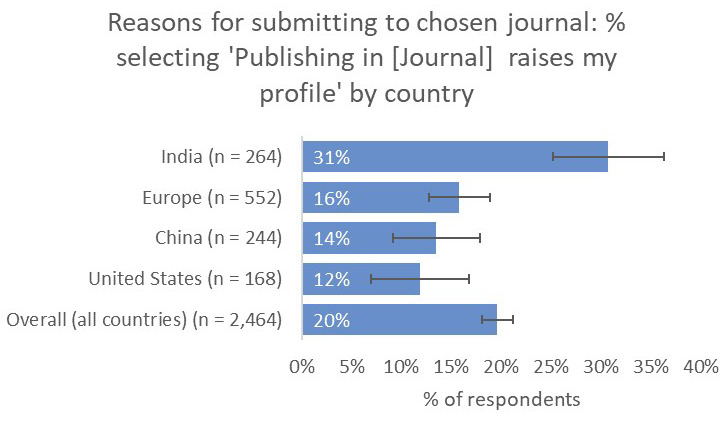
Relative importance of raising the respondent’s profile in determining choice of journal.

Perhaps most importantly, respondents from India indicated that a journal’s capacity to raise their profile was much more important to them (31%) than respondents from the other territories that we considered (UK and Europe 16%, China 14%, US 12%; Figure 17).

### The role of the publisher in driving real-world impact

Academic publishing is multifaceted, with a range of different stakeholders located all around the globe, across both the private and public sectors. We asked participants the following question: *How could journals or publishers help research to influence the response to real-world problems?* Answers were provided as free text and clustered around four main improvements to mechanisms around: access, accessibility, communication of outcomes, and timeliness.

1. Improve access to the latest research, in particular to non-academic/policy-maker audiences, as well as to the underlying code/data.
*“Engage closer with non-governmental organisations (environmental and social) – provide greater access to these organisations that are fundamental to achieving the SDGs but do not have the financial resources to enjoy access/membership of the Journals.”*
*“Provide access to interesting real-world data sets” / “Publish code and data along with papers; special issues focused on practical applications”*

In order to engage a non-academic audience, our respondents’ views are clear: policy-makers, industry, and the wider public must have access to the original research, both the underlying data and the conclusions. In this regard, greater support for open access publication models ^18^ across all key stakeholders is an important step to take to allow non-academic readers to engage with the latest research.

2. Improve accessibility of research by changing the language, style, and format of publications to serve a non-academic audience.
*“Prepare readers’ digest versions of relevant articles, in multiple languages.”*
*“Provide a policy-type document for research papers that tackle real-world problems. Original research paper may be difficult to read by policy-makers.”*
*“Publish an e-digest of abstracts indexed by problem area. Send it to NGOs and managers in government agencies so they can quickly find articles that are relevant for their issues.”*
*“Increased use of executive summaries from research papers that are accessible to a broader audience than academia”*
*“provide support producing infographics and sharing research to non-academic audiences”*

To help realise the potential reach, impact, and policy application of research, respondents noted that research outcomes should be presented in a format, style, and language that is accessible and comprehensible to a non-academic audience. Whilst the research article well-serves the research community, the structure, tone, and length may create some barriers for non-academic readers, who are often looking for evidence pertaining to their particular point of need and may be put-off from drawing out points of relevance from a full research paper.

3. Improve communication links to raise the visibility of research implications on policy and real-world issues.
*“Making more publicity to the "non-scientific world" of the issues that are published in the journals” / “be present at policy events”*
*“Special editions and workshops (can be via Zoom) to bring people together.”*
*“Share published papers on social media and create TV shows where scientists engage on current issues.”*
*“Connections with academic media outlets, like the Conversation etc.”*
*“They should announce research grants related to real world problems”*

Authors and publishers need to maximize the opportunity to bring the latest research into the public conscious, with the aim of cultivating a culture that drives policy change. Respondents noted that non-academic summaries, workshops, and discussion forums could directly engage with policy-makers right at the point of need. However, as noted by one respondent, it is also important for publishers to “be present” where appropriate at policy events and to advocate for the value of the research that they publish on behalf of their authors.

4. Better support the publication of research on areas of particular relevance to live policy issues.
*“Seek out authors who are also practitioners.” / “By opening spaces for discussion among different actors (policy-makers, civil society and academia) and societal sector.”*
*“Be willing to publish applied work, not just academic studies.” / “encourage and publish more transdisciplinary research”*
*“By staying focused on their journals' scope which should be specific to these real-world problems”*
*“By planning special issues which focus on research that are in response to real-world problems. When doing so, ensuring that enough time is given for research in this area to be specifically conducted, and not expecting that data is already available to be tailored into a paper that addresses these issues.”*
*“By considering articles that address real world problems, even it if they are not considered "high impact" or "potentially citable".”*

These comments collectively strike right at the heart of the purpose of research journals.

## Discussion

### The impact gap

Overall, 90% of respondents indicated that their work either currently contributed (directly or indirectly) to meeting real-world problems or that it would be a focus for them in the future. However, when asked about forms of impact, citations were viewed as more important than advancing research or contributing to the SDGs.

We might infer that this focus on citations as the key form of impact is driven by the mechanisms that underpin research assessment. There are calls to move away from a focus on citations and publication venue, and to judge work based on its own merits ^19^. However, changes to institutional assessment mechanisms—and academic culture itself—are slow to take effect. This focus on citations may be compounded by the ways in which impact is judged at a national level, with judgements around research ‘excellence’ by country often based on comparing field-weighted citations from one country to another ^20^.

***Is this an issue worth addressing?*** The answer to this question must be “yes”, both for the research and then for the researcher, institution, funder, and publisher. If the measures that exist within academic publishing remain unchanged, continuing to prioritize other metrics and outcomes, we risk devaluing the necessary application of original research to addressing our global challenges. It is then a slippery slope from devaluing to deprioritising to not doing at all, and the devaluing of important, consequential research today will likely lead to less of it in the years to come, at a time when much more research is required to help meet our global society’s needs, not less.

There is also a cultural issue. If research as a whole does not pursue greater public engagement and support the tackling of our global challenges, the research community risks appearing elitist and out-of-touch with the public conscious ^21^. 

***How might we bridge the gap?*** There is clearly a high level of engagement by the respondents in contributing to the thinking around real-world problems. However, at the heart of academic research, there are seemingly competing interests, which push back on our authors’ desire to tackle the key challenges affecting our society, and instead pull authors to pursue volume of output and accruing citations. Therefore, given this apparent disconnect between the ambitions of researchers to address real-world problems with their work and the realities that drive their choice of journal and preferred type of impact, we next turn to what might be done to bridge this gap.

### Seeing the wood and the trees

There seems to be a knowledge gap for our researchers in understanding and communicating the link between their individual, highly focused projects and wider live policy issues, such as those expressed by the SDGs. Respondents encouraged publishers to facilitate the connection of an individual output to a real-world challenge, such as the SDGs, through the publication of summary research conclusions in approachable language (“lay summaries”, “policy highlights”, or similar), either alongside or as part of the research article, and to work with researchers and institutions to explore new and alternative ways of disseminating such summaries to the general public or a policy audience.

Authors should be encouraged to articulate how their work contributes to real-world challenges, such as how the work aligns with a particular SDG (goal or target). An example of this is the European Commission’s Horizon Results Platform, which allows authors to identify their work with particular SDGs and to flag research outcomes as “Claiming significant policy influence” ^22^. Publishers and journal editors should be proactive in this process through the curation of special issues that are directly linked to live policy-relevant issues, such as those expressed in the Horizon Europe missions, to encourage greater consideration of policy priorities by our researchers. In addition, a focus on interdisciplinarity and the policy relevance of work should be encouraged, as our respondents have indicated that these are essential to ensuring that research has a real-world impact.

To support this work, we will work with our editors and society partners to introduce policy-impact statements widely across our Earth & Environmental Sciences journals over the next 12 months. We hope that this approach will facilitate the contextualisation of new research within larger global needs and aid in the impact of that work on policy decision-making. We will additionally hold a cross-stakeholder discussion forum in 2021 to explore other appropriate structural ways of clarifying policy relevance (such as to particular SDGs) at an article level. We will also publish cross-portfolio special issues on policy-relevant topics, beginning with the policy priorities expressed in the European Commission’s Horizon Europe missions ^23^, to be published on World Earth Day 2022. By doing this, we aim to encourage the greater consideration of policy priorities by our researchers and to support the publication of such research within our journals. Finally, we will continue to transition our Earth & Environmental Sciences journals onto more-open data-sharing policies to support the availability, reuse, and citation of codes and data ^24^.

Institutions could consider developing training programs for their researchers to help them think through how a specialised piece of work can have wider implications, such that it can be incorporated into policy change, and how researchers can communicate and demonstrate the relevance of their work to those discussions.

### Building a pipeline to policy

Comments from respondents suggest that changes to traditional mechanisms around engagement, knowledge transfer, and research assessment are required to create better links and lines-of-sight between research endeavour and policy development. Furthermore, several respondents also felt that addressing real-world problems with their work was not enough in itself to contribute to change; rather, action by policy-makers was a decisive factor in whether their research would have such an influence.

To enthuse researchers with the ambition of addressing real-world impact, they need to become more cognisant of how their research is incorporated into policy advice and decision-making. When asked whether, since the publication of their work and to the best of their knowledge, their article had been used or referenced in a non-academic output, the majority of respondents (32%) answered “don’t know”, and only 10% suggested that their work had been used in a policy document. At present, authors can’t easily track and often aren’t aware if/how their work is used outside of other research articles. Therefore, there needs to be a much-more-robust feedback mechanism from governments, NGOs, lobbyists, and advisory groups to the academic community, e.g. through the expansion of tools such as Altmetric ^25^, if researchers are going to feel sufficiently equipped to engage in policy advocacy and to feel that non-academic outputs, such as those suggested above, would be valued and acted upon.

Universities are well-positioned to educate their faculties about the mechanisms and approaches open to them for communicating their work to a policy audience. It is in the interest of institutions and funders to encourage researchers to engage in policy and advocacy. Research offices can leverage the resources already out there to help with guidance and training, and could consider partnering with groups such as Sense about Science and the UK’s Parliamentary Office on Science & Technology (POST) to share insights from those with close connections to legislators. In collaboration with these groups, Taylor & Francis offers guidance to help published authors on getting their research into the UK Parliament ^26^, as well as supporting the work of Vitae on impact and evaluation ^27^, and we are continually expanding these resources.

Much has been done to support the translation of research conclusions into language suitable for a non-academic audience, through platforms such as Kudos ^28^, or through social media and academic news services such as EurekAlert! ^29^ and the Conversation ^30^. However, such activity has yet to become common practice. The translation of research results into “policymaking language” to make it useable for policy-makers might be achieved through the publication of accompanying abstracts for a non-academic audience or policy implications/highlights for each new piece of work. Ensuring that there are channels for research to reach policymakers is another critical activity publishers should consider. Publishers might consider synthesising and facilitating meta-comparisons of similar research outcomes to support a streamlined evidence-based policymaking process. New products or services could be created to assist in this translation and dissemination activity, as well as the presentation of relevant research to policy-makers and their advisory groups, such as TrendMD ^31^. These services will however require financial support; additionally, researchers need to be incentivised to ensure that they are positively rewarded for making these connections. Such activities must be supported by training and incentives from those host institutions keen to ensure that their research outputs have a real-world impact. To support this activity, publishers and funders should facilitate interoperable standards and persistent identifiers to ensure that all of the outcomes are linked, and that policy decisions are informed by a rich and networked research base.

Most broadly, there may be a need to extend the “research cycle” to incorporate a policy dimension into the initial stage of developing the research question, thereby ensuring that researchers review funding calls in their area and study the research agenda of their governments and policymakers for activities relevant to their field. The European Geosciences Union (EGU) have published a helpful graphic, which outlines the interplay between policy and the research cycle ^32^. Greater adoption of this “policy cycle” approach, including the formalisation of prompts at the dissemination stage of the research cycle to direct relevant outcomes to policy-makers, could help to influence sharing behaviours, and the expansion of a researcher’s existing network into the policy arena.

### Building links and amplifying networks

Publishers might typically express the value that they provide to the academic community through the oversight of rigorous peer-review processes to validate research conclusions and through the curation, dissemination, and preservation in perpetuity of the academic record. However, based on the responses to the questions “Did the publication of your article lead to you forming new connections with any of the following?” and “Have any of the following happened in your career since the publication of your article?”, it is clear that publishers also play a hugely important role in facilitating new collaborations between researchers, which are often triggered by research publications, with collaboration a critical component of research success ^33^. 

In addition, through the promotion and dissemination of their research, publishers have the capability to foster meaningful engagement with the general public through press/news media coverage or mentions on social media. Publishers serve an important role as bridge-builder between communities both within and without academia, and should ensure that they continue in this function.

Respondents raised concerns around working in small silos, with limited personal networks and small spheres of influence, particularly in the policy space, which held back the impact of their research in tacking real-world problems. Our survey highlights some areas where publishers should consider how to better connect researchers with the wider non-academic community to help their work resonate outside academic circles, and to have real-world impact, including network-extending activities with non-academic communities, specifically policy-makers and industry.

We suggest that universities give thought to helping their researchers to better grasp the mechanisms for how their research can influence policy, and what steps they can personally take to advocate for the uptake of their conclusions into the policy debate. Likewise, we encourage funders, universities, and publishers to work with governments and other pertinent stakeholders to develop a more robust feedback mechanism to the academic community so that researchers can understand how their work is used in decision-making and can feel equipped to advocate for a real-world impact with their work.

### Changing the academic currency or research assessment reform

Our feedback suggests that research assessment practices should be reviewed and revised to ensure that policymakers, institutions, and funders capitalise on the aspirations of researchers to effect real-world change and reward those aspirations. Revised practices should be supported by incentives that place greater emphasis on clarifying policy impact in grant applications, and deliver greater recognition for policy impact. In this regard, the Final Report of the Expert Group on Indicators for Researchers’ engagement with open science ^34^, the Open Science Policy Platform’s final report ^35^, and LERU’s Open Science and its role in universities: a roadmap for cultural change report roadmap for institutions ^36^ all include excellent recommendations, and also show how moves to change research assessment practices dovetail with calls to increase open science / research practices.

Our authors expressed a desire to conduct research that helps to tackle global needs, and most are already actively achieving this with their research. However current assessment frameworks continue to focus on and reward citations and publication in highly ranked journals at the expense of opportunities for impact or other forms of output. As part of our ongoing training and support for our authors and editors, we highlight the role of research metrics in measuring performance, but also their limitations, and note that such tools need to be used appropriately, as part of a “basket of metrics”, and not in place of a qualitative review of individual outputs ^37, 38^. We are also investing in diversifying research outputs, including providing support for non-traditional formats, such data notes and software tool articles ^39^. We have committed to making a broad set of metrics available across journals that provide a richer overview of their performance, linked with guidance to contextualise these data.

We suggest that institutions reconsider the performance-assessment frameworks that they use, instead placing greater value on a broader range of research outputs, such as patents, case studies, and engagement with secondary education. Likewise, we suggest that funders continue to facilitate, through the grant applications that they support, the pursuit of research with clearly defined opportunities for policy-relevant outcomes or other tangible real-world impact, such as the Gates Foundation’s support of the Grand Challenges for Global Health (GCGH) initiative ^40, 41^. 

## Conclusion

Following a survey of >2,500 researchers who had published in our Earth & Environmental Sciences journals portfolio, we found that a majority of respondents (90%) indicated that their work either currently contributed to meeting real-world problems or that it would become a priority in the future, thus suggesting that, as one might anticipate, the tackling of real-world challenges is a significant research priority in the Earth & Environmental Sciences.

Whilst it is very encouraging to see that the majority of research in the subject area is concerned (directly or indirectly) with addressing our global needs, the impetus seems to be altruistic researcher desires, rather than incentives or support from publishers, funders, or institutions. As a result, it seems that this laudable ambition is being lost amidst the realities of being a researcher – where success is predominantly measured by citations and publication venue.

Therefore, herein, we have used survey responses to consider what opportunities we have as a research community to collectively assist researchers in having the real-world impact with their work that they (and we) would like it to have. Accompanying this report is a set of suggestions for the wider community, which have been drawn out of the conclusions from this work, along with a series of commitments which we as Taylor & Francis will take to play our part in addressing some of these issues.

We welcome feedback from the community and opportunities for collaboration, and we anticipate that these recommendations will be further refined as we implement our commitments and undergo further consultation.

## Data availability

### Underlying data

Figshare: Taylor-and-Francis_Impact-Assessment-of-Earth-and-Environmental-Sciences-Research-Author-Survey_Raw-Data_Figshare, https://doi.org/10.6084/m9.figshare.13281146.v1 ^42^.

### Extended data

Figshare: Taylor-and-Francis_Earth-and-Environment-Survey-Questions, https://doi.org/10.6084/m9.figshare.13281104.v1 ^13^.

Data are available under the terms of the Creative Commons Attribution 4.0 International license (CC-BY 4.0).

## Author information

Dr Andrew Kelly holds a BSc (First Class Honours) in Chemistry from the University of York (2003) and a PhD in Chemistry from the University of Bath (2008). He has worked in publishing for more than 10 years and is currently a Journals Portfolio Manager at Taylor & Francis, where he is responsible for the Remote Sensing, GIS & Cartography titles. ORCiD: 0000-0002-5785-4782

Victoria Gardner is Director of Policy at Taylor & Francis, where she advises on global policy developments around scholarly communications including around open access, copyright and IP, and the move to digital. She has worked in a variety of roles within Taylor & Francis, including portfolio management, open access, and technology. ORCID: 0000-0002-8519-6377

Anna Gilbert holds a BTh in Theology from Bangor University and an MSc in Social Research Methods from the University of Surrey. She is currently a Research & Analytics Manager at Taylor & Francis, where she is responsible for primary research and marketing/web analytics. ORCiD: 0000-0002-9627-9274
