# The disconnect between researcher ambitions and reality in achieving impact in the Earth & Environmental Sciences – author survey

**DOI:** 10.12688/f1000research.28324.2

**Published:** 2022-07-08

**Authors:** Andrew Kelly, Victoria Gardner, Anna Gilbert

**Affiliations:** 1 Taylor & Francis Group, Abingdon, OX14 4RN, UK

**Keywords:** academic publishing, Earth Sciences, Environmental Sciences, research journals, research assessment, survey, Sustainable Development Goals, SDGs

## Abstract

**Background:** There is an increasing desire for research to provide solutions to the grand challenges facing our global society, such as those expressed in the UN SDGs (“real-world impact”). Herein, we undertook an author survey to understand how this desire influenced the choice of research topic, choice of journal, and preferred type of impact.

**Methods:** We conducted a survey of authors who had published in >100 of our Earth & Environmental Science journals. The survey was sent to just under 60,000 authors and we received 2,695 responses (4% response rate).

**Results:** Respondents indicated that the majority of their research (74%) is currently concerned with addressing urgent global needs, whilst 90% of respondents indicated that their work either currently contributed to meeting real-world problems or that it would be a priority for them in the future; however, the impetus for this research focus seems to be altruistic researcher desire, rather than incentives or support from publishers, funders, or their institutions. Indeed, when contextualised within existing reward and incentive structures, respondents indicated that citations or downloads were more important to them than contributing to tackling real-world problems.

**Conclusions:** At present, it seems that the laudable and necessary ambition of researchers in the Earth & Environmental Sciences to contribute to the tackling of real-world problems, such as those included in the UN SDGs, is seemingly being lost amidst the realities of being a researcher, owing to the prioritisation of other forms of impact, such as citations and downloads.

## Introduction

The role that academic publishing plays in the research process is often understood to comprise registration, curation, evaluation, dissemination, and archiving ^1^. This covers validation (through the peer-review process), publication (participation in the scholarly record), curation (preservation of the work to ensure its availability in perpetuity), and dissemination (to relevant communities). These activities help researchers to advance knowledge by building on existing outcomes, progressing discussion and debate, and driving consensus. However, in the digital age, the potential of a research journal is much broader than this, fostering collaboration, network-building (both within core and adjacent fields), career development, and maximizing the capability of research to mobilise knowledge and contribute to the solving of grand challenges ^2, 3^. Indeed, especially in the Earth and Environmental Sciences, there is increasing pressure on researchers to support policy formulation or to address societal challenges through their research in order to continue to receive research funding ^4^. Therefore, it is essential that the mechanisms and drivers that collectively influence where an author chooses to publish their research support publication in the journals that are most relevant to their work; that is, where their research is most likely to be found, read, cited, and iterated upon by those working in the same and adjacent disciplines, as well as by those working outside of academia, in policy-making, lobbying, or advisory capacities.

However, such mechanisms and drivers, both personal and external, are varied and nuanced, as are our authors’ expectations for what impact that their work might have once it has been published. Academics ^5^, institutions ^6^, publishers ^7^ and learned societies ^8^ often survey their researchers and/or members to understand their values and views towards key issues around topics such as open access, data sharing, reproducibility, and career progression ^9^. Research impact has also been the subject of both surveys ^10^ and research ^11^ in recent years, with common themes emerging around the opportunities presented by the move to digital of open access, and of linking research outputs to broader societal impact or benefit. Furthermore, several national research evaluation systems, such as the UK’s Research Excellence Framework (REF) ^12^ and the Australian Research Council’s Excellence in Research for Australia ^13^ include the potential societal impact of applications in their allocation of research funding. Conversely, common roadblocks have been identified, including access to outputs, incompatible research culture, and an over-emphasis on journal metrics, rather than individual researcher/research-output impact, which lead to a focus on publishing work in highly ranked journals in order to advance in their careers ^5, 14– 16^. For example, one of the main findings from Springer Nature’s 2019 research collaboration on researcher attitudes towards societal impact was the focus from survey respondents on the concept of “academic impact”, which was more important to most respondents than “societal impact beyond academia”.

It is in this context that we undertook the *2020 Impact Assessment of Earth & Environmental Sciences Research: Author Survey*. Surveys have been frequently used for evaluating “research impact” ^17^, and there has been much discussion about the relationship with the UN Sustainable Development Goals (SDGs) within the Earth and Environmental Sciences communities ^18^. Therefore, our survey was particularly designed to achieve three main aims. To understand:

- what drives these communities to choose the topics that they research;
- what drives these communities to choose the journals that they publish in; and
- what type(s) of impact they are most looking for from their work.

We investigated what benefits publishing in our journals could impart on both the research and on the authors following publication, and we looked at to what extent global challenges, such as those expressed in the SDGs and the missions of Horizon Europe, were shaping researcher ambitions.

## Methods

In Spring 2020, Taylor & Francis surveyed authors from across our Earth & Environmental Sciences portfolio. The survey (see *Extended data* ^19^), hosted on Alchemer (formerly SurveyGizmo), was emailed to authors using Salesforce Marketing Cloud. It was sent to just under 60,000 authors and received 2,695 responses (4% response rate).

The survey comprised 23 questions: section A (Q1 & 2) = multiple choice questions to clarify the article that the survey responses related to; section B (Q3 & 4) = multiple choice questions with the option of prose responses relating to the choice of journal; section C (Q5–10) = multiple choice questions with the option of prose responses relating to the downstream value of publishing the article for both the work and the author; section D (Q11–20) = largely multiple choice with the option of prose relating to the impact of the work, the motivation for undertaking the work, and the ability of the work to tackle real-world problems and influence policy change. Questions 13, 15, 18, and 20 were solely prose responses. Section E (Q21–23) = demographic questions.

A confidentiality and privacy statement was provided on the first page of the survey, which outlined how the data would be used. Consent to participate in the survey was implied by the authors who clicked through to complete the questionnaire after reading this statement and the instructions given in the invitation email. The data are fully anonymized and no sensitive personal data regarding the respondents were collected. To protect the anonymity of the respondents, all prose responses to the free-text questions (questions 13, 15, 18, and 20) have been omitted from the shared dataset, though some comments have been included herein. Written informed consent was not sought due to the low-risk nature of the research.

The survey responses include authors from 102 journals in the Earth & Environmental Sciences portfolio, and the geographical distribution of responses was similar to that of authors in the portfolio. Therefore, we can be reasonably confident that our responses are representative of Taylor & Francis authors in our Earth & Environmental Sciences journals.

### Data analysis

Confidence intervals were calculated for certain parts of our analysis in which we compared groups of different sizes, and we are only reporting herein on differences that are statistically significant. Microsoft Excel was used to prepare the tables and charts. Confidence intervals were calculated by using the Creative Research Systems sample-size calculator.

***A note about error bars and statistical significance.*** The country-comparison charts presented in this report include error bars, which plot the confidence intervals for the percentages shown. When making comparisons, error bars are useful as a visual means of demonstrating the range that likely contains the true overall value for each country in the chart. If the error bars for two or more countries overlap, we should be cautious about making substantive conclusions about any differences, because they may not be statistically significant. Therefore, only clearly statistically significant differences are included in the comparisons presented herein.

### Terms and terminology

Herein, we have used a series of terms, including “real-world problems”, “global challenges”, “real-world application”, and “real-world challenges” interchangeably to describe the wider societal impact of a piece of research, which typically occurs downstream of further academic advancements. Research impact, more broadly, incorporates both forms of outputs, and the relationship between a piece of work and these outcomes, for both academic and non-academic stakeholders, is complex ^20, 21^.

## Results and discussion

### SDG-relevance of earth and environment research – the current picture

*‘Why do researchers undertake the research that they do?’* It is a fundamental question and the answer is multifaceted, varying by career stage, geography, and subject discipline. However, the publication of the Sustainable Development Goals (SDGs) by the UN in September 2015, which had the stated aim of providing a “ a shared blueprint for peace and prosperity for people and the planet, now and into the future” , allows us an opportunity to frame the question in such a way that gets to the core of what researchers hope to achieve through their work, that is: ‘ *do researchers study topics that contribute, either directly or indirectly, to the tackling of real-world problems?*’.

**Figure 1.**
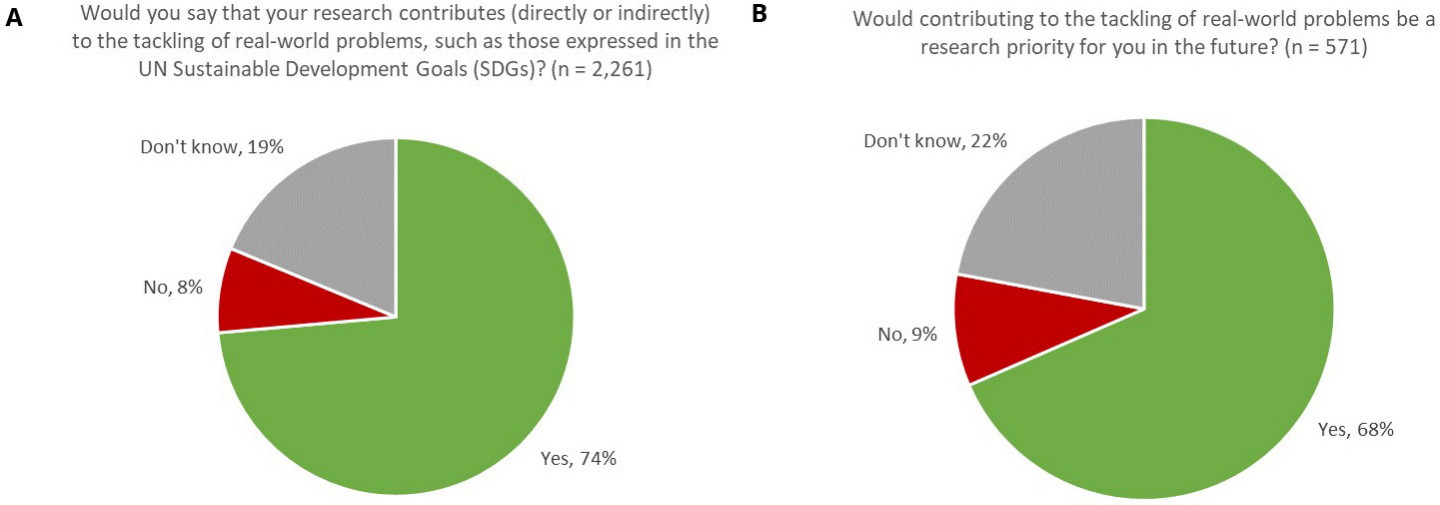
Proportion of respondents whose research contributes to tackling real-world problems, now (A) and in the future (B).

Comprising such urgent needs as Clean Water and Sanitation (SDG 6) and Climate Change (SDG 13), and tackling threats to Life on Land (SDG 15) and Life below Water (SDG 14), one might readily anticipate that a high proportion of research in the Earth and Environmental Sciences would have a part to play in meeting the needs expressed by the SDGs. Indeed, 74% of respondents indicated that, in their opinion and based on their understanding of the broader context of their work and the challenges expressed by the SDGs, their research contributed (directly or indirectly) to the tackling of real-world problems, such as those expressed by the UN SDGs ( Figure 1). Furthermore, 90% of respondents indicated that their work either currently contributed to meeting real-world problems or that it would be a research priority for them in the future. Therefore, we might infer that, in the Earth & Environmental Sciences, it is a strong research imperative for our authors that their work contributes to the tacking of real-world problems.

Such a high percentage aligns with the perspectives of journal editors in these subject areas, who, in contributing to our recent publication *Sustainable Development Goals in the Earth and Environmental Sciences* ^22^, expounded the variety, breadth, and richness of research that their journals and subject areas have to offer in tackling the challenges laid out in the SDGs.

In our survey, whilst younger researchers were slightly more likely to undertake this type of research (76% of respondents aged under 50 answered ‘ *Yes*’ compared with 70% of respondents aged 50 or older, with resolved confidence intervals), the difference was not very pronounced, thus suggesting that this is a multi-generational aspiration, rather than one solely driven by early-career researchers.

Whilst we have noted that the tackling of real-world problems, such as those expressed by the SDGs, was a strong research priority for respondents, 8% of respondents selected ‘ *No*’ when asked if their work had such implications. As a follow-up question to those respondents, we asked the following: *Please could you elaborate on why your research might not necessarily contribute to tackling real-world problems?* and asked for prose responses.

Several responses suggested that their work was too highly specialised, fundamental, narrow in scope, or too preliminary to have broad application to a grand challenge, such as an SDG, whilst others suggested that the contribution of the work depended more on the engagement of policy-makers and aligned government politics than on the relevance of the research. This appears to highlight an opportunity gap around the role that journals could play in maximising the capability of research to mobilise knowledge. This aligns with the view of the International Science Council, noting in its 2021 report that “ *The value of science to national economies and in confronting global challenges demands more efficient processes of knowledge dissemination*” ^23^.

### Comparison of author-led and analytics-led analyses

In addition to qualitative feedback from survey respondents, we also used the Sustainable Development Goals Research Category in Dimensions Analytics to quantitatively analyse the proportion of research published in the surveyed journals that had been linked to one or more of the SDGs ^24^.

To facilitate as close a comparison as possible between the datasets, we analysed the Dimensions records for articles published in the 102 surveyed journals between 2012 and 2020, in line with the distribution list for the survey.

**Figure 2.**
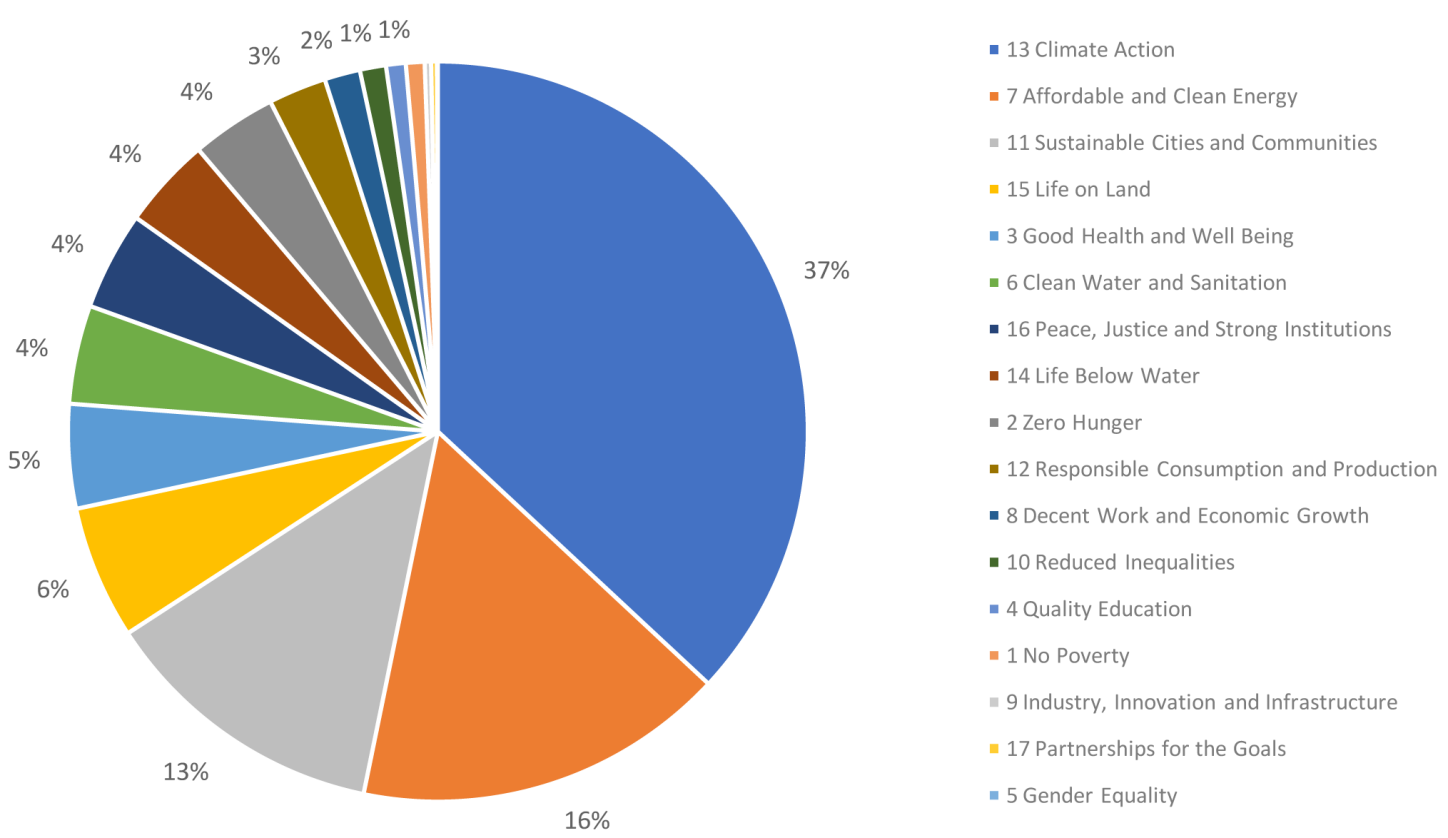
Relative proportion of the different SDGs covered by T&F research corpus (based on number of article tags).

Of the 53,890 published articles, 9,096 (17%) were linked to one or more SDGs, with 10,144 SDG links overall. The relative proportion of article tags to the individual SDGs is shown in Figure 2. As might be expected for Earth and Environmental Sciences subject areas, more than one third of the tagged articles were linked to Climate Action (SDG 13; 37%), followed by Affordable and Clean Energy (SDG 7; 16%) and Sustainable Cities and Communities (SDG 11; 13%).

Notably, the proportion of articles that were linked to one or more SDGs in the Dimensions records (17%) was much lower than the proportion of respondents who indicated that, in their opinion, their research contributed (directly or indirectly) to the tackling of real-world problems (74%). While the survey responses and Dimensions analysis did not overlap entirely in the articles they considered, we think that the overlap was significant enough and the difference was pronounced enough to allow us to make a couple of inferences.

Firstly, the structure of a research article does not typically allow for discussion of the broader implications / applications of a piece of research in contributing to grand challenges, such as the SDGs, which makes meta-analysis more difficult and could mask the relevance of the work to a non-academic audience.

Secondly, the ability of analytics/AI tools to link article-level research with broader problems is improving ^25^, but remains under-developed, and further learning will be required to appropriately characterise research with more-indirect SDG implications.

### Why is addressing real-world challenges a research priority for our authors?

**Figure 3.**
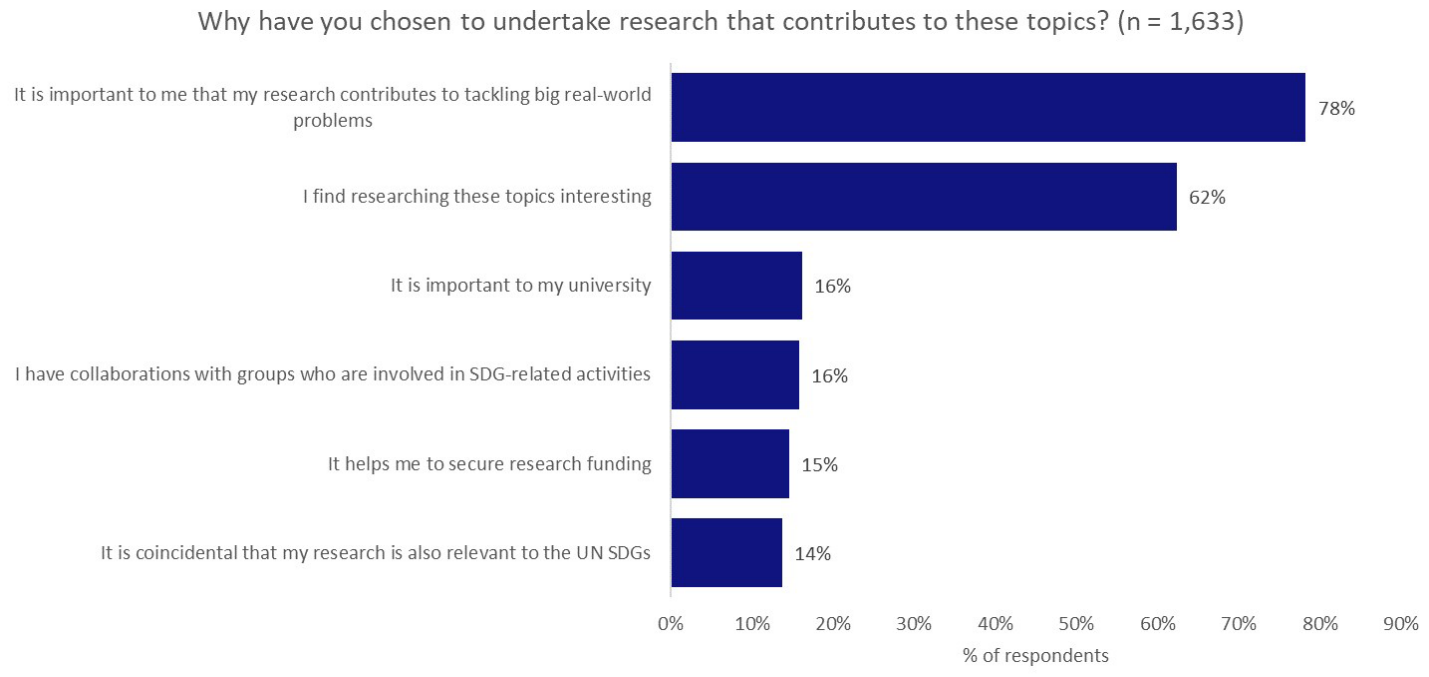
Motivating factors for investigating topics with applications to real-world problems.

To understand a bit more about the motivating factors that sit behind the decision of our researchers to investigate topics that have application to real-world problems, we asked *Why have you chosen to undertake research that contributes to these topics?* ( Figure 3). The responses to this question presented a clear split between internal drivers—personal interest (62%) and the desire to contribute to addressing real-world problems (78%)—and external drivers, such as encouragement from a university (16%), other collaborators (16%), or improved opportunities to secure research funding (15%), with internal drivers and aspirations being much more significant.

We find it surprising that the influence of institutions (16%) and funders (15%) were only narrowly more influential than coincidence (14%) in prompting research that was skewed towards meeting global challenges.

### Ambitions vs reality

**Figure 4.**
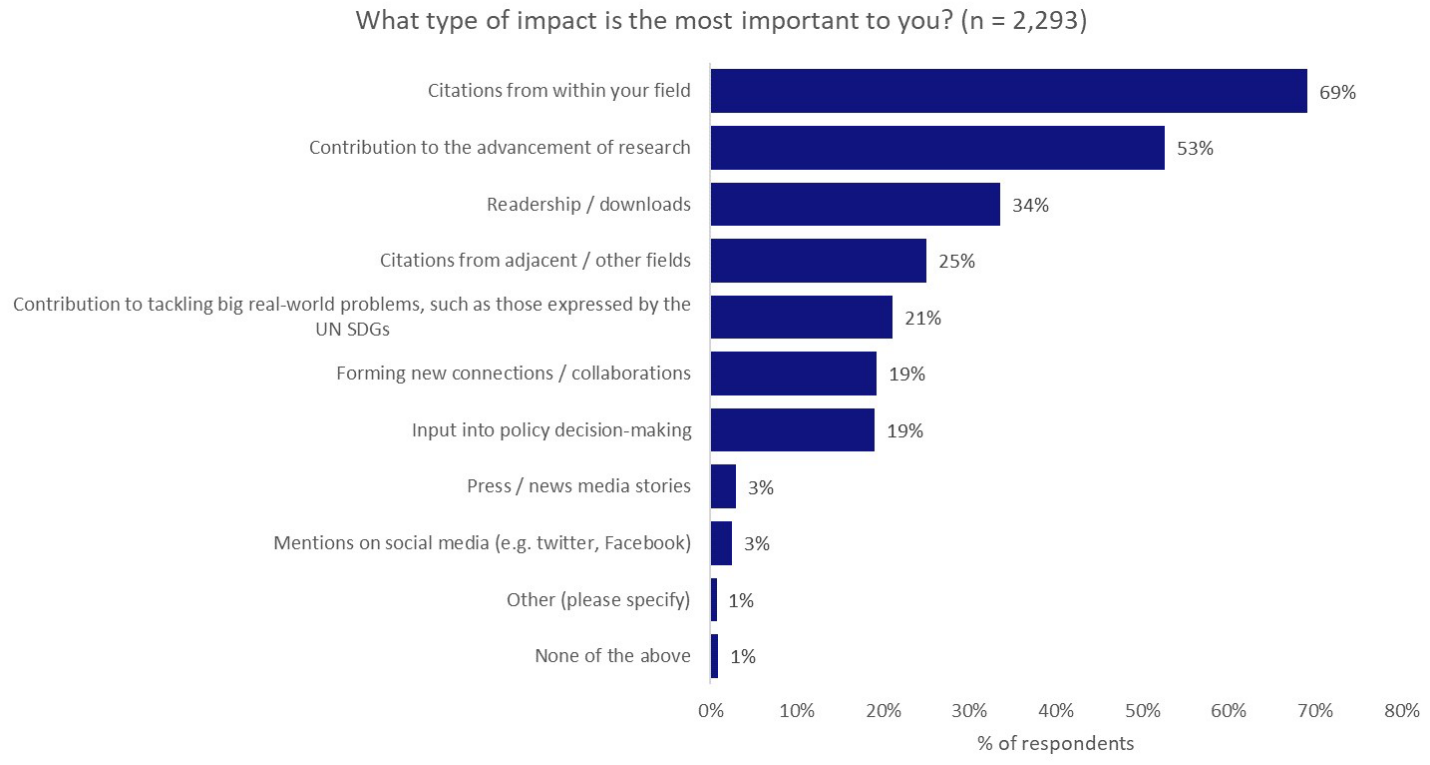
Most-preferred type of impact (maximum of three selections).

We saw the greatest gap between aspiration and reality when we asked what type of impact was most important to our researchers, who were asked to make a maximum of three selections. The most-preferred type of impact was citations from within the same field (69%), coming above contribution to the advancement of research (53%), contribution to tackling real-world problems, such as those expressed by the UN SDGs (21%), and input into policy decision-making (19%; Figure 4).

Interestingly, having seen the strong desire of authors to undertake research with real-world application in the earlier questions, when compared with other types of impact, contributing to the tacking of real-world problems dropped to fifth in the list (21%), behind citations from within the same field (69%) and from adjacent/other fields (25%), and achieving a large readership (34%).

We note that some respondents may have felt that citations were a necessary step in contributing to the advancement of research or in tackling real-world problems, through knowledge sharing and discussion, as the reasons for these selections weren’t probed further. This may be explored further in future research.

Input into policy decision-making, where ideas, research, and theory can be put into practice for much of the national-scale change that is required to meet the needs captured by the UN SDGs (19%), placed further down the list, on par with forming new collaborations (19%). Only attention from the press (3%) and attention on social media (3%) ranked lower.

**Figure 5.**
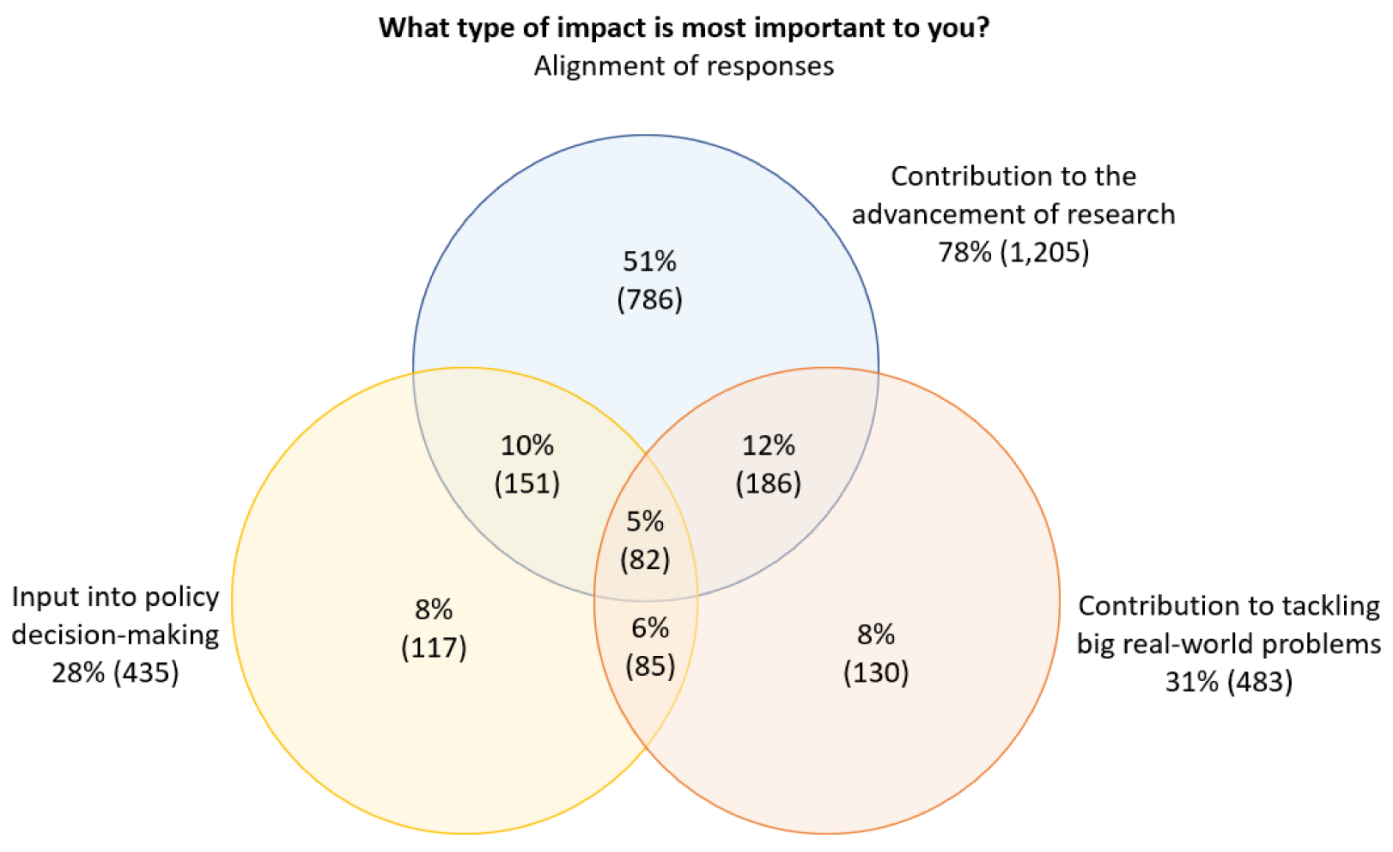
Alignment of selected responses to the question *What type of impact is most important to you?*.

We also considered the extent to which there was overlap between three of the key responses to the question shown in Figure 3: ‘ *contribution to the advancement of research*’, ‘ *contribution to tackling big real-world problems*’, and ‘ *input into policy decision-making*’ ( Figure 5). The percentages shown are based on the total number of respondents who selected at least one of these three options.

Of the respondents who selected one or more of ‘ *contribution to the advancement of research*’, ‘ *input into policy decision-making*’ and ‘ *contribution to tackling big real-world problems*’ as one of their top-three most important types of impact, more than half (51%) only selected ‘ *contribution to the advancement of research*’, which indicated that research impact was much more important than policy and real-world impact.

We also noted modest overlap between respondents who selected either ‘ *input into policy decision-making*’ or ‘ *contribution to tackling big real-world problems*’ and ‘ *contribution to the advancement of research*’, and minor overlap between respondents who selected ‘ *input into policy decision-making*’ and ‘ *contribution to tackling big real-world problems*’.

We did not probe the links between these responses further in the survey, although, as discussed above, several respondents who indicated that their work did not contribute to tackling real-world problems cited the necessary engagement of policy-makers to achieve this form of impact. Furthermore, many of the responses to the question ‘ *How could journals or publishers help research to influence the response to real-world problems?*’ indicated the need to engage a non-academic audience (see below).

### Influence on the choice of journal

**Figure 6.**
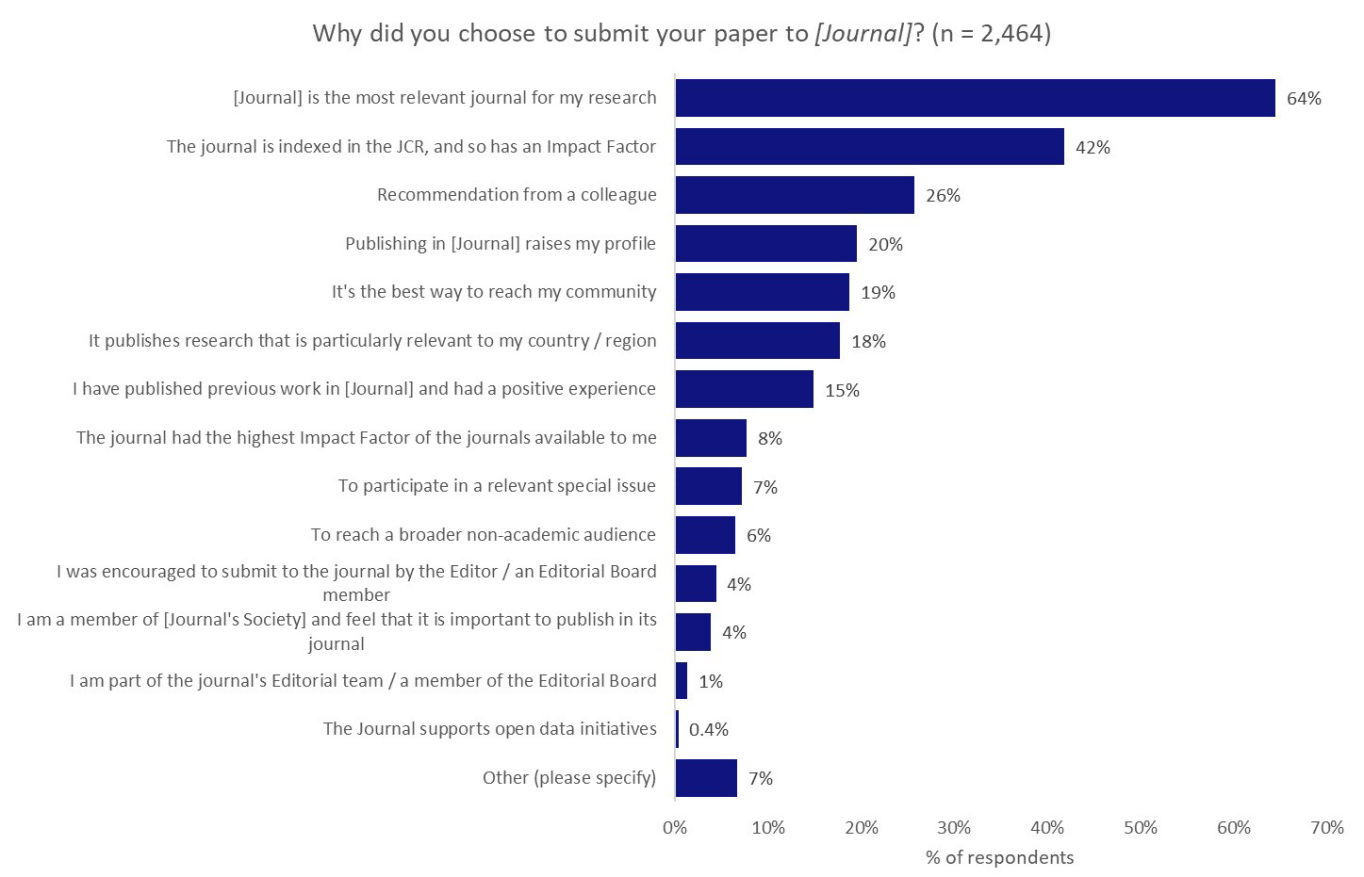
Motivating factors for the choice of journal.

Feedback from respondents has indicated two key points: 1) the aspiration of researchers to contribute to the tackling of real-world problems with their work; and 2) an emphasis on citations and readership as primary measures of impact ^26, 27^. We wanted to understand what role the choice of journal played in meeting researchers’ aspirations. To investigate this question, and to see if there might be a correlation between the choice of journal and their preferred type of impact, we asked all of the respondents why they chose to submit their paper to the journal that their work was published in ( Figure 6).

The predominant factor in determining the selection of a journal was its relevance to the author’s research, and 64% of respondents indicated this was one of the most-influential factors underpinning their choice of journal. Reaching a broader non-academic audience (which we might link to the desire to contribute to resolving real-world challenges) came quite far down the list, with only 6% of respondents noting that this was an influential factor in determining their choice of journal.

Interestingly, although 42% of authors indicated that the journal having an Impact Factor was an important factor in their decision-making, only 8% indicated that they chose the journal because it had the highest available Impact Factor, perhaps indicating that the presence of an Impact Factor was more important than the score itself. Many institutions, policy-makers, and funders are keen to reduce emphasis on the Impact Factor as part of research assessment practices ^28^, so there is perhaps a misalignment in the priorities of researchers, as opposed to their institutions and funders.

We also asked whether the respondents had published in their first-choice journal to understand the possible clouding of a correlation between the preferred type of impact and choice of journal by not being able to publish in their first choice. We found that 75% of respondents indicated that they published in their first-choice journal, whilst 19% indicated that it was their second choice and 6% indicated that they had previously submitted their work to more than one other journal. Based on these responses, we inferred that it was reasonable to make the correlation between citation as the preferred form of impact and the importance of a journal receiving an Impact Factor.

### Comparing priorities across different geographies

Scholarly communication is multifaceted, with a range of different stakeholders located all around the globe, across both the private and public sectors. Indeed, we found that authors located in different regions placed different emphases on the criteria that shaped their choice of journal, and their preferred types of impact. We discuss below findings from some specific countries or regions with noteworthy responses, and have made all findings available in the supplementary dataset.

**Figure 7.**
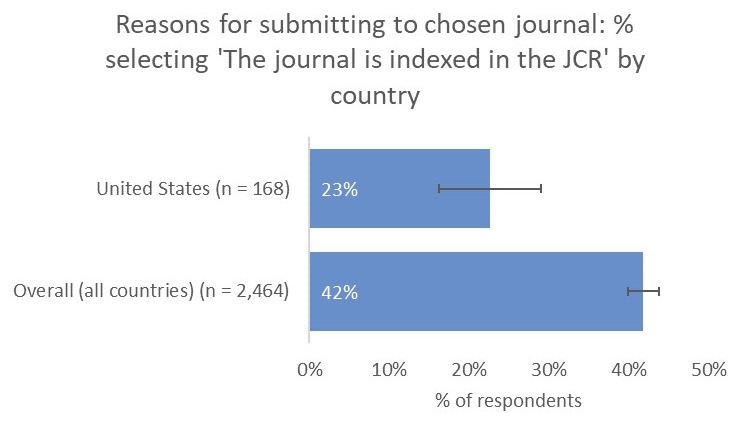
Relative importance of indexing in the JCR in determining choice of journal for US-based authors.

***United States.*** Fewer respondents based in the United States indicated that having an Impact Factor was an important criterion in determining their choice of journal compared to the overall average (23% vs 42; Figure 7). Of note, this feedback corresponded with the results of our post-publication author survey ^29^, which is sent to authors in all subject areas and all geographies. Respondents from the US typically rate that having an Impact Factor is a less-important factor in determining their choice of journal compared with the global average.

**Figure 8.**
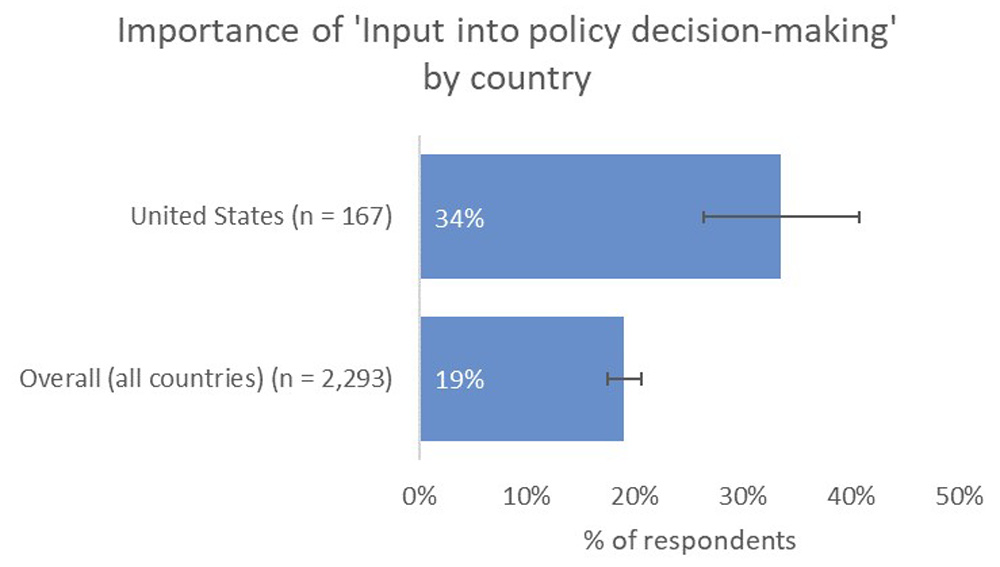
Relative importance of input into decision making for US-based authors.

Instead, US-based authors placed more value on real-world types of impact than the global average, with a higher proportion indicating that contribution to tackling big real-world problems, such as those expressed by the UN SDGs, was one of the most-important types of impact to them (29%), and a much-higher proportion indicating that having an input into policy decision-making was important to them (34% vs 19% overall; Figure 8).

**Figure 9.**
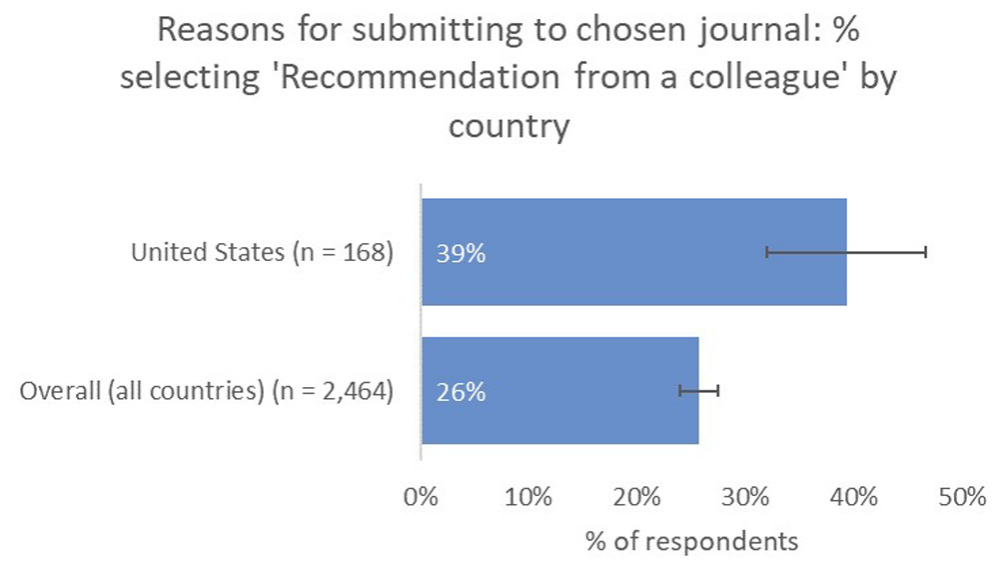
Relative importance of recommendation from a colleague in determining choice of journal for US-based authors.

Recommendation from a colleague (39% vs 26% overall) and the journal’s capacity to reach a broader non-academic audience (13% vs 7% overall) were also deemed to be much more important factors as a means of identifying a suitable journal for US-based respondents, compared to the global average ( Figure 9).

***China.*** Responses from researchers based in China largely reflected the overall results, both in the most-important types of impact to them and the most-important factors that influence their choice of journal. As in other geographies, respondents from China indicated that receiving citations from within the same field was one of the most-important types of impact for them (72%), followed by contribution to the advancement of research (49%) and readership/downloads (33%).

**Figure 10.**
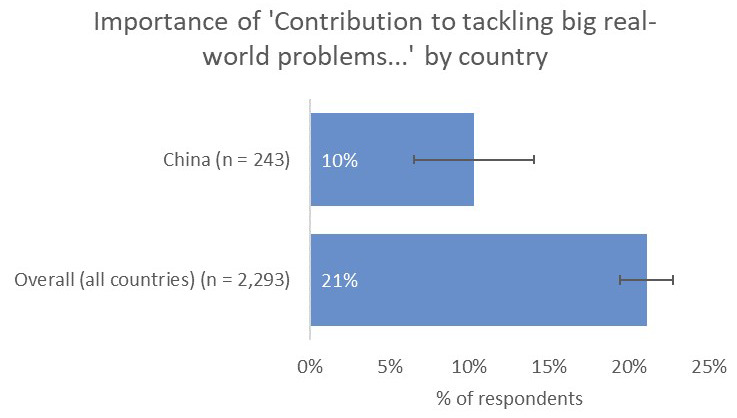
Relative importance of tackling real-world problems to China-based authors.

**Figure 11.**
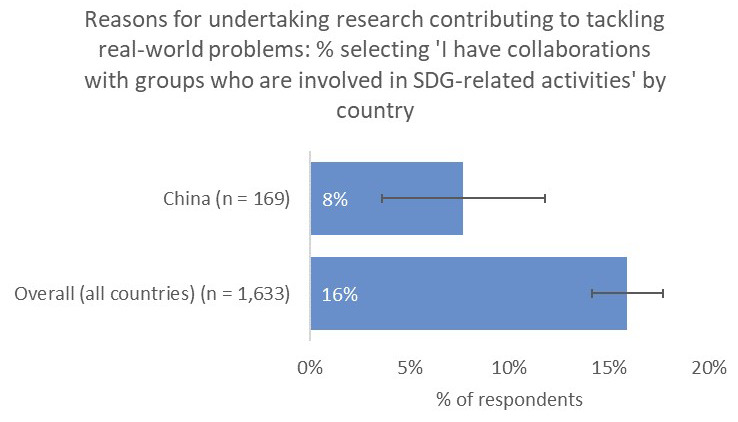
Relative proportion of researchers with collaborators who are involved in SDG-related activities.

However, one noticeable distinction was the relative unimportance of having a real-world impact in terms of contribution to tackling real-world problems (10% vs 21% overall; Figure 10) and input into policy decision making (8% vs 19% overall), perhaps because China-based respondents were less likely to have collaborations with groups who were involved in SDG-related activities (8% vs 16% overall; Figure 11).

**Figure 12.**
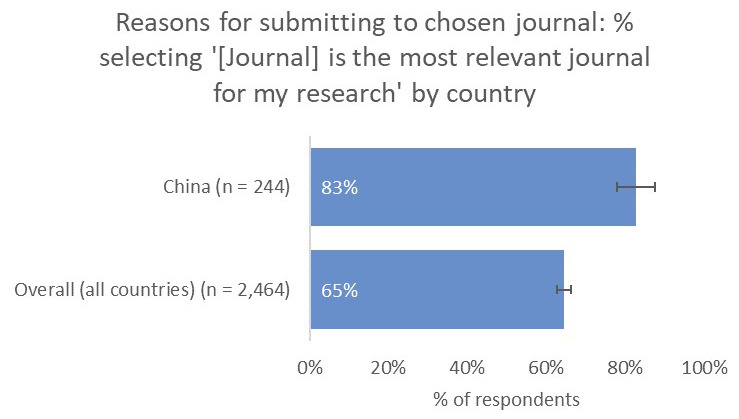
Relative importance of a journal’s relevance in determining choice of journal for China-based authors.

Conversely, for China-based researchers, the relevance of a journal to their work was a much-more-important consideration (83%) compared to the global average (65%) and compared to authors based in the US (59%) and Europe (49%), whilst whether the journal had an Impact Factor was as important to Chinese respondents (44%) as the overall average (42%; Figure 12).

***Europe (including the UK).*** European respondents closely followed the global averages for both the types of impact that were most important and the most-important factors in determining the choice of journal.

**Figure 13.**
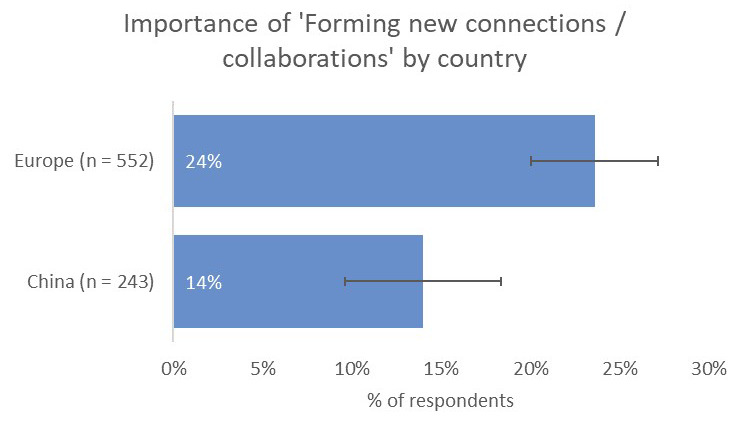
Relative importance of new collaborations as a form of impact for Europe- and China-based authors.

Respondents indicated that receiving citations from within the same field was the most-important type of impact to them (73%), followed by contribution to the advancement of research (50%) and readership/downloads (33%). Where respondents based in Europe differed was in the prospect of forming new collaborations (24%), which they considered to be a more-important type of impact than for respondents from India (16%) and China (14%; Figure 13).

**Figure 14.**
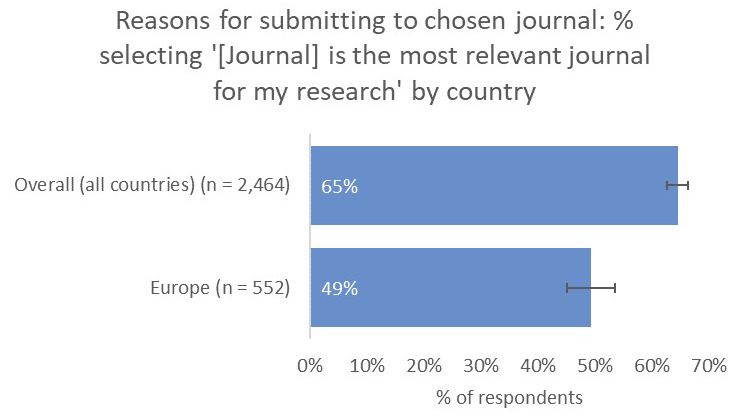
Relative importance of relevance in determining choice of journal for Europe-based authors.

Interestingly, and perhaps related to the premium placed on network-building outside of their own subject communities, only 49% of respondents from Europe said that the relevance of the journal to their work was a key factor in influencing their choice of publication venue, much lower than all other territories (65% average; Figure 14).

***India.*** Respondents from India again closely followed the global averages in terms of the types of impact that were most important and the most-important factors in determining the choice of journal. However, there were two distinct points of divergence.

**Figure 15.**
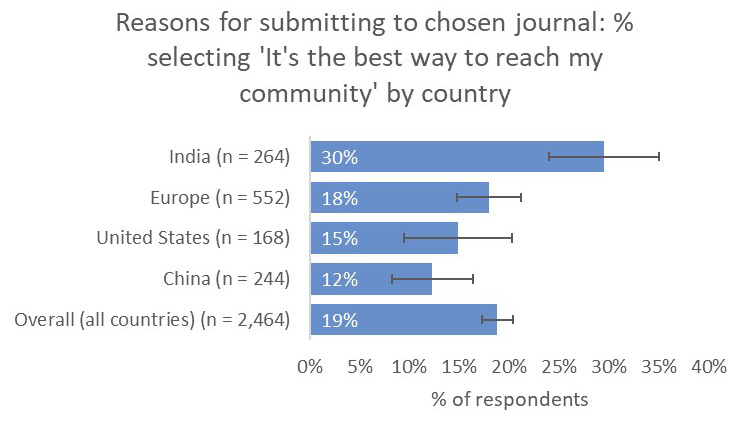
Relative importance of reaching a target community between the demographics considered.

Compared to the global average, respondents from India placed significantly greater importance on the relevance of the journal for their work (87% vs 65% overall), comparable to respondents from China (83%) and significantly higher than respondents from the US (59%) and the UK and Europe (49%). Similarly, respondents from India placed much greater importance on the journal’s capability to reach their community (30%) compared to respondents from China (12%), the US (15%), and UK and Europe (18%), as well as to the overall average (19%; Figure 15).

**Figure 16.**
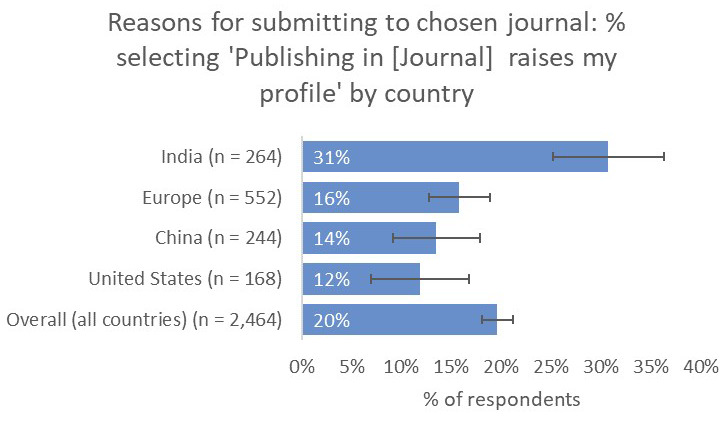
Relative importance of raising the respondent’s profile in determining choice of journal.

Perhaps most importantly, respondents from India indicated that a journal’s capacity to raise their profile was much more important to them (31%) than respondents from the other territories that we considered (UK and Europe 16%, China 14%, US 12%; Figure 16).

### Driving real-world impact

Finally, we asked participants the following question: *How could journals or publishers help research to influence the response to real-world problems?* Answers were provided as prose and our analysis found suggestions clustered around four main improvements to mechanisms around: access, accessibility, communication of outcomes, and timeliness, as described below.

**1.**  Improve access to the latest research, in particular to non-academic/policy-maker audiences, as well as to the underlying code/data.
*“Engage closer with non-governmental organisations (environmental and social) – provide greater access to these organisations that are fundamental to achieving the SDGs but do not have the financial resources to enjoy access/membership of the Journals.”*
*“Provide access to interesting real-world data sets” / “Publish code and data along with papers; special issues focused on practical applications”*
**2.**  Improve the accessibility of research by changing the language, style, and format of publications to serve a non-academic audience.
*“Prepare readers’ digest versions of relevant articles, in multiple languages.”*
*“Provide a policy-type document for research papers that tackle real-world problems. Original research paper may be difficult to read by policy-makers.”*
*“Publish an e-digest of abstracts indexed by problem area. Send it to NGOs and managers in government agencies so they can quickly find articles that are relevant for their issues.”*
*“Increased use of executive summaries from research papers that are accessible to a broader audience than academia”*
*“provide support producing infographics and sharing research to non-academic audiences”*
**3.**  Improve communication links to raise the visibility of research implications on policy and real-world issues.
*“Making more publicity to the "non-scientific world" of the issues that are published in the journals” / “be present at policy events”*
*“Special editions and workshops (can be via Zoom) to bring people together.”*
*“Share published papers on social media and create TV shows where scientists engage on current issues.”*
*“Connections with academic media outlets, like the Conversation etc.”*
*“They should announce research grants related to real world problems”*
**4.**  Better support the publication of research on areas of particular relevance to live policy issues.
*“Seek out authors who are also practitioners.” / “By opening spaces for discussion among different actors (policy-makers, civil society and academia) and societal sector.”*
*“Be willing to publish applied work, not just academic studies.” / “encourage and publish more transdisciplinary research”*
*“By staying focused on their journals' scope which should be specific to these real-world problems”*
*“By planning special issues which focus on research that are in response to real-world problems. When doing so, ensuring that enough time is given for research in this area to be specifically conducted, and not expecting that data is already available to be tailored into a paper that addresses these issues.”*
*“By considering articles that address real world problems, even it if they are not considered "high impact" or "potentially citable".”*

In order to engage a non-academic audience, the respondents indicated that policy-makers, industry, and the wider public must have access to the original research, both the underlying data and the conclusions. In this regard, greater support for open research, such as greater provision of open access publication models across all key stakeholders, could be an important step to take to allow non-academic readers to access and engage with the latest research.

To help realise the potential reach, impact, and policy application of the latest original research, respondents noted that research outcomes should be presented in a format, style, and language that is accessible and comprehensible to a non-academic audience ^30^, or to pursue tailored research syntheses for a particular point of use, although such syntheses have been found to have varied impacts on policy and practice ^31^. Whilst the research article well-serves the research community, the structure, length, and tone may create some barriers for non-academic readers, who are often looking for evidence pertaining to their particular point of need and may be put-off from drawing out points of relevance from a full research paper.

Finally, respondents indicated that authors and publishers should seek to maximize the opportunity to bring the latest research into the public conscious, with the aim of cultivating a culture that drives policy change by engaging with live policy issues. It was suggested that this could be achieved by adopting a transdisciplinary approach at the outset of a piece of work, involving scientific and societal stakeholders ^32^. Respondents noted that non-academic summaries, workshops, and discussion forums could directly engage with policy-makers right at the point of need. However, as noted by one respondent, it is important for publishers to “be present” where appropriate at policy events and to advocate for the value of the research that they publish on behalf of their authors. Such value, which increasingly extends to public and policy engagement must also be recognised and valued by institutions and funders with respect to career advancement and reputational growth ^33, 34^.

## Conclusion

Following a survey of >2,500 researchers who had published in our Earth & Environmental Sciences journals portfolio, we found that a majority of respondents (90%) indicated that their work either currently contributed to meeting real-world problems or that it would become a priority in the future, thus suggesting that, as one might anticipate, the tackling of real-world challenges is a significant research priority in the Earth & Environmental Sciences.

Whilst it is encouraging to see that the majority of research in the subject area is concerned (directly or indirectly) with addressing global needs, the impetus seems to be altruistic researcher desire, rather than incentives or support from publishers, funders, or institutions. As a result, it seems that this necessary application of original research is being lost amidst the realities of being a researcher – where success is predominantly measured by citations and readership. Respondents suggested four key areas for action by publishers and other stakeholders across the scholarly communication ecosystem to help researchers meet their aspiration for their work to have real world impact: access, accessibility, communication of outcomes, and timeliness.

## Data availability

### Underlying data

Figshare: Taylor-and-Francis_Impact-Assessment-of-Earth-and-Environmental-Sciences-Research-Author-Survey_Raw-Data_Figshare, https://doi.org/10.6084/m9.figshare.13281146.v1 ^35^.

### Extended data

Figshare: Taylor-and-Francis_Earth-and-Environment-Survey-Questions, https://doi.org/10.6084/m9.figshare.13281104.v1 ^19^.

Data are available under the terms of the Creative Commons Attribution 4.0 International license (CC-BY 4.0).

This paper was written using data obtained on (DATE), from Digital Science’s Dimensions platform, available at https://app.dimensions.ai. Access was granted to subscription-only data sources and functions under licence agreement.

https://doi.org/10.6084/m9.figshare.20176412

## Author information

Dr Andrew Kelly holds a BSc (First Class Honours) in Chemistry from the University of York (2003) and a PhD in Chemistry from the University of Bath (2008). He has worked in publishing for more than 10 years and is currently a Journals Portfolio Manager at Taylor & Francis, where he is responsible for the Remote Sensing, GIS & Cartography titles. ORCiD: 0000-0002-5785-4782

Victoria Gardner is Director of Policy at Taylor & Francis, where she advises on global policy developments around scholarly communications including around open access, copyright and IP, and the move to digital. She has worked in a variety of roles within Taylor & Francis, including portfolio management, open access, and technology. ORCID: 0000-0002-8519-6377

Anna Gilbert holds a BTh in Theology from Bangor University and an MSc in Social Research Methods from the University of Surrey. She is currently a Research & Analytics Manager at Taylor & Francis, where she is responsible for primary research and marketing/web analytics. ORCiD: 0000-0002-9627-9274
