# Supplementary material for: The disconnect between researcher ambitions and reality in achieving impact in the Earth & Environmental Sciences – author survey
Source: F1000Res. 2023 Mar 3;10:36. Originally published 2021 Jan 19. [Version 3] doi: 10.12688/f1000research.28324.3 (PMC10076906; doi:10.12688/f1000research.28324.3)
Supplement: Peer review history

# Peer review: Reviewer response for version 3

**DOI:** 10.5256/f1000research.145008.r165356

**Published:** 2023-04-05

**Authors:** Kathryn Oliver

**Affiliations:** 1 Faculty of Public Health and Policy, London School of Hygiene and Tropical Medicine, London, UK

Hallo, thanks for your detailed responses which address all my main points. Thanks also for the HEPI note and related links which I enjoyed reading.

Is the work clearly and accurately presented and does it cite the current literature?

Yes

If applicable, is the statistical analysis and its interpretation appropriate?

Yes

Are all the source data underlying the results available to ensure full reproducibility?

Yes

Is the study design appropriate and is the work technically sound?

Yes

Are the conclusions drawn adequately supported by the results?

Yes

Are sufficient details of methods and analysis provided to allow replication by others?

Yes

Reviewer Expertise:

Evidence production and use, research impact, academic-policy engagement

I confirm that I have read this submission and believe that I have an appropriate level of expertise to confirm that it is of an acceptable scientific standard.

# Peer review: Reviewer response for version 2

**DOI:** 10.5256/f1000research.57459.r161241

**Published:** 2023-02-24

**Authors:** Kathryn Oliver

**Affiliations:** 1 Faculty of Public Health and Policy, London School of Hygiene and Tropical Medicine, London, UK

**Thanks for a chance to read this paper, from an interesting mixed team. Apologies from me too that this has taken so long to deliver; I can see that the first revision has already produced lots of improvement.**

 Back in 2014 I undertook a systematic review of barriers and facilitators of evidence use in policy. Most of the 145 studies included were surveys of academics by other academics, identifying factors which they felt affected whether or not their research had impact ^1^.

 I mention this because as far as we could tell, these surveys were nearly all based on researchers’ opinions, which could not be verified from observational, ethnographic or experimental studies of evidence use in policy. This doesn’t imply that researchers are providing misleading answers, but there is a well-documented comprehension gap in academic generally about how policy works and how research impact actually occurs.

 You deal with this a bit in the ambitions vs. reality section, where you contrast the stated desire of researchers to undertake applied research with their preference for citations. You could discuss this a bit more by explaining what you (and if possible they) mean by ‘real world application’ and the other categories. I suspect most researchers think that their work does in some way ‘tackle’ a real world problem - the question is at how many degrees removal is that the case. Overall I would urge caution in how interpretations of this finding is worded, e.g. researchers “indicated that they BELIEVED their work either currently contributed to meeting real-world problems or that it would become a priority in the future”, and  “Whilst it is encouraging to see that RESEARCHERS FEEL THAT the majority of research in the subject area is concerned (directly or indirectly) with addressing global needs” etc.

 For me the most interesting bit is about the role of journals and publishers. There is some connection here to the history of learned societies, who as you know were the first publishers of regular academic communications ^2^^,^^3^. The suggestions raised by your respondents are all very instrumental (e.g. formatting etc) but I wonder if you would like to comment on the role of journals in promoting relationships and community, in raising the quality of research (through forging those links) and maintaining standards (which of course could include utility of research as a quality marker).

 Some of the other suggestions raised by respondents (e.g. more academic-policy engagement) relate to an evidence base which is actually pretty weak. Some of the data we collected in this project ^4^ looked at publisher- and journal-led activities to promote research impact, although we didn’t find many examples. Happy to share our data on this if helpful - I think there were a couple of prizes offered by publishers (e.g. Emerald) and some convening.

Is the work clearly and accurately presented and does it cite the current literature?

Yes

If applicable, is the statistical analysis and its interpretation appropriate?

Yes

Are all the source data underlying the results available to ensure full reproducibility?

Yes

Is the study design appropriate and is the work technically sound?

Yes

Are the conclusions drawn adequately supported by the results?

Yes

Are sufficient details of methods and analysis provided to allow replication by others?

Yes

Reviewer Expertise:

Evidence production and use, research impact, academic-policy engagement

I confirm that I have read this submission and believe that I have an appropriate level of expertise to confirm that it is of an acceptable scientific standard, however I have significant reservations, as outlined above.

## References

[1] : A systematic review of barriers to and facilitators of the use of evidence by policymakers. *BMC Health Serv Res* .2014;14: 10.1186/1472-6963-14-2 2 10.1186/1472-6963-14-2 24383766PMC3909454

[2] : The role of learned societies in national scholarly publishing. *Learned Publishing* .2020;33(1) : 10.1002/leap.1270 5-13 10.1002/leap.1270

[3] : The role of learned societies in knowledge exchange and dissemination: the case of the Regional Studies Association, 1965–2005. *History of Education* .2011;40(2) : 10.1080/0046760X.2010.518161 255-271 10.1080/0046760X.2010.518161

[4] : What works to promote research-policy engagement?. *Evidence & Policy* .2022; 10.1332/174426421X16420918447616 1-23 10.1332/174426421X16420918447616

# Response

**Published:** 2023-02-28

**Authors:** Andrew Kelly

**Affiliations:** Taylor & Francis Group, UK

*Thanks for a chance to read this paper, from an interesting mixed team. Apologies from me too that this has taken so long to deliver; I can see that the first revision has already produced lots of improvement.*

 We’re grateful for you making the time to review our paper and for your comments.

*Back in 2014 I undertook a systematic review of barriers and facilitators of evidence use in policy. Most of the 145 studies included were surveys of academics by other academics, identifying factors which they felt affected whether or not their research had impact.*

*I mention this because as far as we could tell, these surveys were nearly all based on researchers’ opinions, which could not be verified from observational, ethnographic or experimental studies of evidence use in policy. This doesn’t imply that researchers are providing misleading answers, but there is a well-documented comprehension gap in academic generally about how policy works and how research impact actually occurs.*

*You deal with this a bit in the ambitions vs. reality section, where you contrast the stated desire of researchers to undertake applied research with their preference for citations. You could discuss this a bit more by explaining what you (and if possible they) mean by ‘real world application’ and the other categories. I suspect most researchers think that their work does in some way ‘tackle’ a real world problem - the question is at how many degrees removal is that the case.*

 A similar point was raised by another reviewer, and we agree that we likely presumed too much understanding of this term by the respondents to the survey, which may have been exacerbated by our use of a series of terms interchangeably without definition. The 2014 article is well-noted, thank you for highlighting it. In the first revision, we added a section, Terms and terminology, to briefly define our understanding of these terms, and we have now added a comment that these terms may not have been commonly understood by all respondents to the survey, which may have then contributed to the gap between our authors’ feedback and the Dimensions analysis. We have also expanded this section to further articulate what we understand by these terms.

*Overall I would urge caution in how interpretations of this finding is worded, e.g. researchers “indicated that they BELIEVED their work either currently contributed to meeting real-world problems or that it would become a priority in the future”, and  “Whilst it is encouraging to see that RESEARCHERS FEEL THAT the majority of research in the subject area is concerned (directly or indirectly) with addressing global needs” etc.*

 Yes, this is a fair criticism. We have revised some of our terminology to be more dispassionate, rather than inferring the feelings/beliefs of our respondents.

*For me the most interesting bit is about the role of journals and publishers. There is some connection here to the history of learned societies, who as you know were the first publishers of regular academic communications. The suggestions raised by your respondents are all very instrumental (e.g. formatting etc) but I wonder if you would like to comment on the role of journals in promoting relationships and community, in raising the quality of research (through forging those links) and maintaining standards (which of course could include utility of research as a quality marker).*

 Our author survey also included questions relating to the extent to which the article influenced future career events for our authors (e.g., future collaboration, promotion, or invitation to join an Editorial Board/speak at a conference), and, as you mention, whether publication of their article led to the forming of new connections (both within the subject area and with non-academic communities, and domestically/internationally). We have omitted these results from this paper, as we felt that they related more to the wider value of publishing in academic journals, rather than the main topic of this paper, but would be happy to share the data with you and have expanded on this slightly.

*Some of the other suggestions raised by respondents (e.g. more academic-policy engagement) relate to an evidence base which is actually pretty weak. Some of the data we collected in this project looked at publisher- and journal-led activities to promote research impact, although we didn’t find many examples. Happy to share our data on this if helpful - I think there were a couple of prizes offered by publishers (e.g. Emerald) and some convening.*

 Thank you for sharing this paper. We have included a comment to note some caution about the effectiveness of such engagement activities. Yes please, we would be very interested in discussing your analysis and picking this discussion up separately as we look to develop future activities in this area.

 Since this survey, we have been working on the topic of engagement and impact, with a focus on the research-policy exchange, and published a Policy Note with the UK Higher Education Policy Institute (HEPI) on this topic last year, which we’d be delighted to discuss with you in more detail (available here: https://www.hepi.ac.uk/wp-content/uploads/2022/12/Why-open-access-is-not-eno.ugh-Spreading-the-benefits-of-research-1.pdf).  You may also find a recent report from HEPI of interest as well: ‘How to talk to policymakers about research’, based on interviews with those experienced in working in the policy and HE sectors: https://www.hepi.ac.uk/wp-content/uploads/2023/01/How-to-talk-to-policymakers-about-research.pdf.

# Peer review: Reviewer response for version 2

**DOI:** 10.5256/f1000research.57459.r143684

**Published:** 2022-07-11

**Authors:** Brooks Hanson

**Affiliations:** 1 American Geophysical Union, Washington, USA

The authors have revised the paper extensively and seem to have at least considered all the original comments constructively. The paper is now "published".

 My only comment is wrt ref. 29. Does F1000 allow references to "data not available?" This seems odd for a journal demonstrating otherwise complete transparency. I understand that the full author survey is not available, but it would seem that the authors/TF could at least extract these data if they want to cite it and provide basic statistics around it (N of respondents, etc.)

Is the work clearly and accurately presented and does it cite the current literature?

No

If applicable, is the statistical analysis and its interpretation appropriate?

Yes

Are all the source data underlying the results available to ensure full reproducibility?

Yes

Is the study design appropriate and is the work technically sound?

Partly

Are the conclusions drawn adequately supported by the results?

Partly

Are sufficient details of methods and analysis provided to allow replication by others?

Yes

Reviewer Expertise:

Earth and space science broadly, and scholarly publishing

I confirm that I have read this submission and believe that I have an appropriate level of expertise to confirm that it is of an acceptable scientific standard.

# Response

**Published:** 2023-02-28

**Authors:** Andrew Kelly

**Affiliations:** Taylor & Francis Group, UK

We have prepared a summary sheet of the data for this question, which has been hosted on FigShare, and updated the reference. Thank you for raising this issue.

# Peer review: Reviewer response for version 1

**DOI:** 10.5256/f1000research.31330.r79623

**Published:** 2021-04-12

**Authors:** Martin Dominik

**Affiliations:** 1 Centre for Exoplanet Science, School of Physics & Astronomy, University of St Andrews, North Haugh, UK

I am having some difficulties with the policy framing of the article, and it does not become obvious what point exactly the authors intend to make. A few statements don’t appear to match up.

 The article touches on many topics, but I feel that none of them are discussed to a sufficient extent. It initially centres around 3 key questions that are to be addressed by a survey of authors, but towards the end it morphs into an essay on developing an environment that supports translating research into policy, which is not much underpinned by the survey data. Consequently, the article feels like two that are loosely connected. Moreover, it sometimes reads like a policy statement and advertisement by Taylor & Francis rather than a research article. This impression is strengthened by the lack of research articles amongst the references in conjunction with the authors not adequately positioning their findings in the context of other research on the topic.

 There is a fundamental conflict between the aspiration of “maximising the capability of research to achieve” and focusing efforts on addressing urgent needs, which remains unresolved in the article. Throughout, these are conflated and confused. I would consider “capability” a most relevant keyword in this context.

 Several references are ill-chosen for supporting the specific point that the authors try to make. Specifically, the Lisbon strategy (reference 1) has the declared aim “to make Europe the most competitive and dynamic knowledge-based economy in the world, capable of sustainable economic growth with more and better jobs and greater social cohesion”, but it does not include an explicit call for research to have “impact”. Moreover, the report of reference 21 states that “People are broadly split on whether the UK invests too much in long-term R&D rather than solving issues that matter now (33% agree vs. 35% disagree)”. The link in reference 20 does not work.

 It is rather unusual for me to defend Michael Gove, but “we no longer need experts” is a (popular) misquotation by omission. He stated: “I think the people of this country have had enough of experts with organisations with acronyms saying that they know what is best and getting it consistently wrong.”, and the latter part of the statement is much relevant for scientists engaging in public debate. It is important to know how to build and maintain trust.

 While the authors elaborate on the term “impact”, it remains problematic and likely to be understood in various ways. One could challenge the statement about quantification in economic terms being straightforward, and in particular question whether benefits should be evaluated in such a way. For example, people dying early could be economically beneficial, but would that be societally desired?

 The “missions” approach in Horizon Europe is somewhat controversial. Notably, the recent 2020 Euroscience Open Forum (ESOF) included a session “Does science for missions undermine the missions of science?”. Likewise, the authors state that the drive to solve "the Grand Challenges of our time" has acquired increased urgency during the COVID-19 pandemic, but one could also argue that prominently reveals a potential flaw of focusing on identified challenges, which is in neglecting those strands of research that are most suitable to provide the basis for the next challenges that we are to encounter (e.g. https://www.statnews.com/2020/02/10/fluctuating-funding-and-flagging-interest-hurt-coronavirus-research/ and https://www.nytimes.com/2021/04/08/health/coronavirus-mrna-kariko.html)

 The authors refer to citations as the primary currency of success or progress, but it might be worth keeping in mind that it is a widespread myth that this applies universally. In particular, the quoted UK Research Excellence Framework (REF) is not based on citation counts. Moreover, there are significant differences between “impact” in the REF and “Pathways to impact” in the context of funding applications to UK Research Councils. Both are distinct from the “Pathways to Impact initiative” (reference 11). I appreciate the authors mentioning a “chain reaction” emerging from original research. Could one elaborate on what would determine the value of research?

 With regard to the role of academic publishing, I note that the International Science Council has recently published an insightful report “Opening the record of science: making scholarly publishing work for science in the digital era.” ^1^

 The definition of the three main aims of the survey does not specify what group of people “our communities” refers to. I am far less confident than the authors about the respondents being “representative”. I would expect that those who just care about their bibliometric profile and other similar performance indicators are not inclined to spend any time responding, which would result in the respondents being more engaged for the scientific community and the wider society.

 I found the “note about error bars and statistical significance” almost entirely stating trivialities, whereas the authors do not provide the crucial information of what the quoted “error bars” actually refer to and what they mean, which leave me unable to interpret them.

 There are some substantial weaknesses with the survey questions and the provided answer options. It is somewhat confusing that in some cases respondents were able to pick any number of answers from a list, whereas in others the number of choices was limited. This poses some difficulties for the interpretation of the results and the limitations to answer options should be mentioned clearly in the respective figure and/or captions. It would also be useful if the authors referred to the question numbers.

 My main concern with regard to the survey is about Q14 and Q16, which refer to “real-word problems”. I think that it is an unfortunate choice that the authors put these central rather than referring to Q11 in conjunction with Q3 on addressing the question on why researchers undertake the research that they do. While the term “real-world problem” carries a polemic tone suggesting that academics might be detached from reality, “directly or indirectly contributing” is remarkably fuzzy. It is not clear to me what Q14 and Q16 are actually able to capture, and I feel that answering with “yes”, “no”, or “Don’t know” is mostly a matter of interpretation of the question. I could make a case for my research falling into either of these categories, depending on what point of view I assume. In fact, “don’t know” appears to be a good option given that for some research the connection to “real-word problems” is not immediately apparent and the connection might only be built in the future. Apparently, a substantial number of respondents chose that option. I also note that Q16 refers to “priority” whereas Q14 does not. I did not see the authors commented on lower numbers for an affirmative response on Q16 as compared to Q14.

 I also wonder how many of the respondents are familiar with what the UN SDGs are, or are willing to look at up before they answer the question. I note that SDG 8 explicitly recognises creativity and innovation as drivers of economic growth, which aligns fundamental research, not directly targeted at specific challenges, with the UN SDGs.

 Something that puzzles me is that 38% of the respondents did not choose the answer “I find researching these topics interesting”. Why do they do research that they are not interested in?

 It would seem to me that the survey reveals another “gap” than the one the authors claim. A key gap appears to be in how the research actually materialises into something useful, with only about 20% of the survey respondents stated that “input into policy decision-making” or “contribution to tackling big real-world problems, such as those expressed by the UN SDGs” was amongst the most important forms of “impact” (although they were limited to 3 answers). In contrast, the authors elaborate on the point that respondents ranked formal recognition over making a contribution and state that we risk devaluing the necessary application of original research to addressing our global challenges by prioritising other metrics and outcomes. However, their study does not provide evidence for that. If the research of the respondents is oriented towards “real-world problems”, the underlying motivation is not the relevant issue. Unfortunately, the authors do not elaborate on to what extent “contribution to the advancement of research” is aligned with “contribution to tackling real-world problems” and/or “input into policy decision-making” or rather not. It is the more unfortunate that respondents were restricted to a maximum of 3 answers for Q11 rather than being able to state where each of them ranks in priority.

 On the question of why authors chose to submit their manuscript to the specific journal, the top chosen answer is pretty much an umbrella category that encompasses more than half of the other answer options, which are more specific on what most “relevant” means.

 The authors should define what they consider “Europe”, e.g. if respondents stated that they are located in Turkey, have their answers been included or not? To my knowledge, the UK is in Europe.

 The mentioned effort on narrowing the science-policy gap is laudable, but can we expect getting the researchers onboard?

 The authors mention “traditional” mechanisms around engagement, knowledge transfer, and research assessment, but in particular with respect to the latter, there are only fashions, but no tradition. A tradition only gets established once something is passed on from generation to generation, while we saw substantial changes on shorter time-scales. Notably, the h-index was not invented before 2005.

 The authors argue that universities are well-positioned to “educate” their faculties, but are we facing a question of education? If researchers are motivated, aren’t they in need of support rather than incentives?

 Channels to reach policy makers is certainly a relevant point, but looking at social media and news services, the question of quality pops up. Scientists with a large public followership are not necessarily the best suited to speak on a specific topic.

Is the work clearly and accurately presented and does it cite the current literature?

No

If applicable, is the statistical analysis and its interpretation appropriate?

No

Are all the source data underlying the results available to ensure full reproducibility?

Yes

Is the study design appropriate and is the work technically sound?

Partly

Are the conclusions drawn adequately supported by the results?

No

Are sufficient details of methods and analysis provided to allow replication by others?

No

Reviewer Expertise:

Astronomy, science policy

I confirm that I have read this submission and believe that I have an appropriate level of expertise to state that I do not consider it to be of an acceptable scientific standard, for reasons outlined above.

## References

[1] : Opening the record of science: making scholarly publishing work for science in the digital era. *International Science Council* .2021; 10.24948/2021.01 10.24948/2021.01

# Response

**Published:** 2022-06-30

**Authors:** Andrew Kelly

**Affiliations:** Taylor & Francis Group, UK

Reponses have been added in-line below the reviewer's comments and are shown in *italics*.

 Reviewer's comments:

 I am having some difficulties with the policy framing of the article, and it does not become obvious what point exactly the authors intend to make. A few statements don’t appear to match up.

 The article touches on many topics, but I feel that none of them are discussed to a sufficient extent. It initially centres around 3 key questions that are to be addressed by a survey of authors, but towards the end it morphs into an essay on developing an environment that supports translating research into policy, which is not much underpinned by the survey data. Consequently, the article feels like two that are loosely connected. Moreover, it sometimes reads like a policy statement and advertisement by Taylor & Francis rather than a research article. This impression is strengthened by the lack of research articles amongst the references in conjunction with the authors not adequately positioning their findings in the context of other research on the topic.

*We agree that the article needed to be more focused and has been reframed around the results of the author survey, rather than the non-citation value of academic research. The policy framing has been removed.*

 There is a fundamental conflict between the aspiration of “maximising the capability of research to achieve” and focusing efforts on addressing urgent needs, which remains unresolved in the article. Throughout, these are conflated and confused. I would consider “capability” a most relevant keyword in this context.

 Several references are ill-chosen for supporting the specific point that the authors try to make. Specifically, the Lisbon strategy (reference 1) has the declared aim “to make Europe the most competitive and dynamic knowledge-based economy in the world, capable of sustainable economic growth with more and better jobs and greater social cohesion”, but it does not include an explicit call for research to have “impact”. Moreover, the report of reference 21 states that “People are broadly split on whether the UK invests too much in long-term R&D rather than solving issues that matter now (33% agree vs. 35% disagree)”. The link in reference 20 does not work.

 It is rather unusual for me to defend Michael Gove, but “we no longer need experts” is a (popular) misquotation by omission. He stated: “I think the people of this country have had enough of experts with organisations with acronyms saying that they know what is best and getting it consistently wrong.”, and the latter part of the statement is much relevant for scientists engaging in public debate. It is important to know how to build and maintain trust.

*Yes, this section was initially reframed to focus on the increased public trust in science/research during the pandemic, but has now been removed owing to the tighter focus and the references have been updated.*

 While the authors elaborate on the term “impact”, it remains problematic and likely to be understood in various ways. One could challenge the statement about quantification in economic terms being straightforward, and in particular question whether benefits should be evaluated in such a way. For example, people dying early could be economically beneficial, but would that be societally desired?

*We agree that the difficulty in qualifying impact itself is part of the challenge, whilst quantification isn’t necessarily a good thing. We have reframed around the mobilisation/transfer of knowledge.*

 The “missions” approach in Horizon Europe is somewhat controversial. Notably, the recent 2020 Euroscience Open Forum (ESOF) included a session “Does science for missions undermine the missions of science?”. Likewise, the authors state that the drive to solve "the Grand Challenges of our time" has acquired increased urgency during the COVID-19 pandemic, but one could also argue that prominently reveals a potential flaw of focusing on identified challenges, which is in neglecting those strands of research that are most suitable to provide the basis for the next challenges that we are to encounter (e.g.

 https://www.statnews.com/2020/02/10/fluctuatingfunding-and-flagging-interest-hurt-coronavirus-research/ and https://www.nytimes.com/2021/04/08/health/coronavirus-mrna-kariko.html)

*Yes, we agree, although the missions concept is gaining traction worldwide, potentially at the expense of curiosity-driven research. The discursive elements around Horizon Europe has been removed as part of the tightening of the article.*

 The authors refer to citations as the primary currency of success or progress, but it might be worth keeping in mind that it is a widespread myth that this applies universally. In particular, the quoted UK Research Excellence Framework (REF) is not based on citation counts. Moreover, there are significant differences between “impact” in the REF and “Pathways to impact” in the context of funding applications to UK Research Councils. Both are distinct from the “Pathways to Impact initiative” (reference 11). I appreciate the authors mentioning a “chain reaction” emerging from original research. Could one elaborate on what would determine the value of research?

 With regard to the role of academic publishing, I note that the International Science Council has recently published an insightful report “Opening the record of science: making scholarly publishing work for science in the digital era.”1

*Thank you for sharing the reference. Our experience suggests that this is the case, along with other examples, such as Horizon Europe’s business case, which comments on increased citations comparatively, but this has been modified or removed in the Introduction.*

 The definition of the three main aims of the survey does not specify what group of people “our communities” refers to. I am far less confident than the authors about the respondents being “representative”. I would expect that those who just care about their bibliometric profile and other similar performance indicators are not inclined to spend any time responding, which would result in the respondents being more engaged for the scientific community and the wider society.

*We also agree that survey sampling tends to lead to some degree of self-selection, which may emphasise some biases. However, we were satisfied that the total number of responses across a wide range of journals and the geographical alignment of the respondents with the journals’ author base allowed us to have reasonable confidence in the representative nature of the results.*

 I found the “note about error bars and statistical significance” almost entirely stating trivialities, whereas the authors do not provide the crucial information of what the quoted “error bars” actually refer to and what they mean, which leave me unable to interpret them.

*The error bars plot the confidence intervals for the percentages shown. If the error bars for two or more countries overlap, we have been cautious about making any substantive conclusions, because they may not be statistically significant, and only clearly statistically significant differences are discussed in our comparisons.*

 There are some substantial weaknesses with the survey questions and the provided answer options. It is somewhat confusing that in some cases respondents were able to pick any number of answers from a list, whereas in others the number of choices was limited. This poses some difficulties for the interpretation of the results and the limitations to answer options should be mentioned clearly in the respective figure and/or captions. It would also be useful if the authors referred to the question numbers.

*The format of the question was selected according to the purpose of the question and the number of perceived answers. The phrasing of the questions was appropriate for the settings that were used; that is, the questions that presented a limited number of options used wording that emphasised priority, whereas the questions that allowed for an unlimited number of selections used wording that emphasised relevancy.*

*With regards the question that allowed a maximum of three responses, this was labelled clearly within the paper. within the text and chart labels.*

 My main concern with regard to the survey is about Q14 and Q16, which refer to “real-word problems”. I think that it is an unfortunate choice that the authors put these central rather than referring to Q11 in conjunction with Q3 on addressing the question on why researchers undertake the research that they do. While the term “real-world problem” carries a polemic tone suggesting that academics might be detached from reality, “directly or indirectly contributing” is remarkably fuzzy. It is not clear to me what Q14 and Q16 are actually able to capture, and I feel that answering with “yes”, “no”, or “Don’t know” is mostly a matter of interpretation of the question. I could make a case for my research falling into either of these categories, depending on what point of view I assume. In fact, “don’t know” appears to be a good option given that for some research the connection to “real-word problems” is not immediately apparent and the connection might only be built in the future. Apparently, a substantial number of respondents chose that option. I also note that Q16 refers to “priority” whereas Q14 does not. I did not see the authors commented on lower numbers for an affirmative response on Q16 as compared to Q14.

*We used the phrase “real-world problems” as we felt it was commonly used parlance in aggregating topics such as the SDGs, but agree that there is some subjectivity there and there is a need for education of researchers in contextualising their work. We have included a brief section to introduce the terms below the note on error bars.*

 I also wonder how many of the respondents are familiar with what the UN SDGs are, or are willing to look at up before they answer the question. I note that SDG 8 explicitly recognises creativity and innovation as drivers of economic growth, which aligns fundamental research, not directly targeted at specific challenges, with the UN SDGs.

*Whilst we did not probe the degree of familiarity of the respondents with the UN SDGs, or the scope of individual Goals or Targets, we believe that, especially in the subject areas covered by the survey, it is reasonable to assume that most respondents are broadly familiar with the priorities of the SDGs and that respondents who felt they were not sufficiently familiar with the SDGs to answer the question would have answered “don’t know”.*

 Something that puzzles me is that 38% of the respondents did not choose the answer “I find researching these topics interesting”. Why do they do research that they are not interested in?

 It would seem to me that the survey reveals another “gap” than the one the authors claim. A key gap appears to be in how the research actually materialises into something useful, with only about 20% of the survey respondents stated that “input into policy decision-making” or “contribution to tackling big real-world problems, such as those expressed by the UN SDGs” was amongst the most important forms of “impact” (although they were limited to 3 answers).

*Thank you for sharing this observation, which has been included in the revision.*

 In contrast, the authors elaborate on the point that respondents ranked formal recognition over making a contribution and state that we risk devaluing the necessary application of original research to addressing our global challenges by prioritising other metrics and outcomes. However, their study does not provide evidence for that. If the research of the respondents is oriented towards “real-world problems”, the underlying motivation is not the relevant issue. Unfortunately, the authors do not elaborate on to what extent “contribution to the advancement of research” is aligned with “contribution to tackling real-world problems” and/or “input into policy decision-making” or rather not. It is the more unfortunate that respondents were restricted to a maximum of 3 answers for Q11 rather than being able to state where each of them ranks in priority.

*We have added a new Venn diagram (Figure 5), which looks at the overlap of responses to three of the key options from question 11. The percentages are based on the total number of respondents selecting at least one of these three options. We chose to limit the number of responses to Q11 because we felt that, whilst it would have been worthwhile asking respondents to rank all of the answers, it would have been a much-larger undertaking to ask respondents to rank or rate nine answers. Additionally, asking respondents to rank all of the answers would not have allowed them to rank things as equally important/unimportant, or to leave some items unranked.*

 On the question of why authors chose to submit their manuscript to the specific journal, the top chosen answer is pretty much an umbrella category that encompasses more than half of the other answer options, which are more specific on what most “relevant” means.

 The authors should define what they consider “Europe”, e.g. if respondents stated that they are located in Turkey, have their answers been included or not? To my knowledge, the UK is in Europe. The mentioned effort on narrowing the science-policy gap is laudable, but can we expect getting the researchers onboard?

*A mapped list of countries to the regions that were used in the analysis has been added to the FigShare deposit.*

 The authors mention “traditional” mechanisms around engagement, knowledge transfer, and research assessment, but in particular with respect to the latter, there are only fashions, but no tradition. A tradition only gets established once something is passed on from generation to generation, while we saw substantial changes on shorter time-scales. Notably, the h-index was not invented before 2005.

 The authors argue that universities are well-positioned to “educate” their faculties, but are we facing a question of education? If researchers are motivated, aren’t they in need of support rather than incentives?

 Channels to reach policy makers is certainly a relevant point, but looking at social media and news services, the question of quality pops up. Scientists with a large public followership are not necessarily the best suited to speak on a specific topic.

# Peer review: Reviewer response for version 1

**DOI:** 10.5256/f1000research.31330.r77703

**Published:** 2021-02-10

**Authors:** Brooks Hanson

**Affiliations:** 1 American Geophysical Union, Washington, USA

Review of Andrew *et al.*, The disconnect between researcher ambitions and reality in achieving impact in the Earth & Environmental Sciences – narrowing the gap.

 This is a report of an interesting survey getting at a major question related to understanding motivations of researchers in conducting and publishing research, and ultimately how to align incentives to support, recognize, and reward better research and activities of scholars aimed at addressing societal challenges and working with communities.

**Major points:**

 1) The main improvement needed for this paper is placing it in context of other work and author surveys. There are many related and similar surveys and analyses, done by publishers, societies, funders, and scholars, and none (not exaggerating; none) are cited or mentioned. Much of the findings here regarding citations and priorities around publishing, open access, and more, have been covered in other recent author surveys, in this general discipline and other disciplines. This context is essential for this paper to be considered scholarly (and published in a scholarly journal). I’ve reviewed a lot of papers over the years, and this is the first submitted to a leading journal where I’ve seen such a lack of referencing. This might be acceptable for a report by a publisher (cf. Elsevier’s recent gender analysis, self-published, also completely without references) but not a submission to a scholarly journal. Most of the references are just to websites, not any formal survey results or scholarly research on these topics (there’s a lot even in the past few years). Such comparisons would also strengthen some of the conclusions.

 Just a note that JpGU and AGU have conducted a somewhat similar survey of their members. The results are not published yet but were presented in this session: https://agu.confex.com/agu/fm20/meetingapp.cgi/Session/105702 at the recent AGU Fall Meeting (see presentation starting at about 40 minutes; registration is required). I’ve been involved in helping this survey. Overall these results (and others AGU has conducted but not published) are similar to the results given in the later questions here regarding selecting journals, citations, etc. However, on the motivation for research (first question in this survey, and the one that sets that main stage for discussion), the AGU-JpGU wording was different but a large number of respondents, well beyond a majority, indicated that their primary motivation for research was around basic “discovery” or “elaboration/synthesis” rather than “responding to responsibility of society.” JpGU members even moreso. In this survey, unlike the T&F one, there was a large age-related difference between early career, and later-career respondents (early career researchers were more focused on topics related to societal impact). I suspect that the populations of respondents overlap heavily in the two surveys. Recognizing that the AGU-JpGU results are not yet fully analyzed or published, I’m just raising this to bring caution to over interpreting the first question of this survey as worded. This question is, however, the most interesting one to explore and provides much of the interesting novelty here. Just be cautious in interpreting the answers.

 One test would be also to simply score recent publications (outputs) as to whether they align with the results—that is, do most of the outputs directly or indirectly support SDGs, for example? My sense is that in the Earth and space sciences, many indirectly do, but that the path is long and I’m not sure 75% would without quite a stretch.

 2) As the authors note there is a disconnect between authors reporting that they are working (directly or indirectly) on societal relevant topics vs. the “impact” (that is, citations) that they are seeking in their work. Further exploration is needed on whether the respondents misinterpreted the first question or if it was worded so vaguely (“indirectly”) as to be meaningless. Note that much “basic” research in the Earth, environmental, and space science has widespread indirect impacts. Much real time “basic-science” data about the Earth is used in the GPS system, weather predictions, or other uses, e.g. For examples, see this discussion here that I was involved with: https://eos.org/editors-vox/earth-and-space-science-for-the-benefit-of-humanity and the linked papers. Indeed many grant applications require a statement regarding impacts.

 Similarly, results for the question on impact expected by the authors are used in comparison. I also wonder if the wording and reality of the scope of published papers drove this response (that is, many have an indirect vs. direct impact) and the response was viewed as a direct impact.

 3) The authors list a number of actions T&F are taking or should take. Interestingly, T&F has not signed DORA—as Springer-Nature and Elsevier have now signed (whatever one thinks of that), Wiley and T&F are the major publishers who have not (many individual society journals published with Wiley have). Perhaps the authors could indicate why or why not that would be appropriate and how to leverage that impact. Here’s a recent editorial from a T&F publication: https://www.tandfonline.com/doi/full/10.1080/10919392.2018.1522774

 4) The authors indicate what some stakeholders, especially publishers, might do. In the Earth environment and space sciences, there are several leading global societies. These are not mentioned. What is their role? Many have missions aligned with providing benefits to society and many are involved in science communication, policy, outreach and training/mentoring (moreso than most commercial publishers and indeed universities). Indeed this might be an argument to focus on publishing with a society versus a commercial title, where these resources are more directly leveraged.

**Other items:**

 The authors argue that it is surprising that JIF is important to researchers but that they don’t always/regularly choose the highest JIF journals when submitting. This is because researchers know rejection rates and do optimization around likelihood of success (or they don’t want to waste their time, which is also important).

Is the work clearly and accurately presented and does it cite the current literature?

No

If applicable, is the statistical analysis and its interpretation appropriate?

Yes

Are all the source data underlying the results available to ensure full reproducibility?

Yes

Is the study design appropriate and is the work technically sound?

Partly

Are the conclusions drawn adequately supported by the results?

Partly

Are sufficient details of methods and analysis provided to allow replication by others?

Yes

Reviewer Expertise:

Earth and space science broadly, and scholarly publishing

I confirm that I have read this submission and believe that I have an appropriate level of expertise to state that I do not consider it to be of an acceptable scientific standard, for reasons outlined above.

# Response

**Published:** 2022-07-01

**Authors:** Andrew Kelly

**Affiliations:** Taylor & Francis Group, UK

Responses to the reviewer comments have been added in *italics*.

 Reviewer's comments:

 This is a report of an interesting survey getting at a major question related to understanding

 motivations of researchers in conducting and publishing research, and ultimately how to align

 incentives to support, recognize, and reward better research and activities of scholars aimed at addressing societal challenges and working with communities.

**Major points:**

 1) The main improvement needed for this paper is placing it in context of other work and author surveys. There are many related and similar surveys and analyses, done by publishers, societies, funders, and scholars, and none (not exaggerating; none) are cited or mentioned. Much of the findings here regarding citations and priorities around publishing, open access, and more, have been covered in other recent author surveys, in this general discipline and other disciplines. This context is essential for this paper to be considered scholarly (and published in a scholarly journal).

 I’ve reviewed a lot of papers over the years, and this is the first submitted to a leading journal where I’ve seen such a lack of referencing. This might be acceptable for a report by a publisher (cf. Elsevier’s recent gender analysis, self-published, also completely without references) but not a submission to a scholarly journal. Most of the references are just to websites, not any formal survey results or scholarly research on these topics (there’s a lot even in the past few years). Such comparisons would also strengthen some of the conclusions.

 Just a note that JpGU and AGU have conducted a somewhat similar survey of their members. The results are not published yet but were presented in this session:

 https://agu.confex.com/agu/fm20/meetingapp.cgi/Session/105702 at the recent AGU Fall Meeting (see presentation starting at about 40 minutes; registration is required). I’ve been involved in helping this survey.

*Thank you and we acknowledge the limitations of our introduction. As part of the refocusing of the article, we have pared-back the introduction to the article and included additional referencing to support the discussion.*

 Overall these results (and others AGU has conducted but not published) are similar to the results given in the later questions here regarding selecting journals, citations, etc. However, on the motivation for research (first question in this survey, and the one that sets that main stage for discussion), the AGU-JpGU wording was different but a large number of respondents, well beyond a majority, indicated that their primary motivation for research was around basic “discovery” or “elaboration/synthesis” rather than “responding to responsibility of society.” JpGU members even moreso. In this survey, unlike the T&F one, there was a large age related difference between early career, and later-career respondents (early career researchers were more focused on topics related to societal impact). I suspect that the populations of respondents overlap heavily in the two surveys. Recognizing that the AGU-JpGU results are not yet fully analyzed or published, I’m just raising this to bring caution to over interpreting the first question of this survey as worded. This question is, however, the most interesting one to explore and provides much of the interesting novelty here. Just be cautious in interpreting the answers.

*Thank you for raising this and we agree that it would be interesting to investigate further.*

 One test would be also to simply score recent publications (outputs) as to whether they align with the results—that is, do most of the outputs directly or indirectly support SDGs, for example? My sense is that in the Earth and space sciences, many indirectly do, but that the path is long and I’m not sure 75% would without quite a stretch.

*This was a very interesting suggestion. We have used Dimensions SDGs category data (new Figure 2) to analyse their quantitative alignment of research published in the same set of journals with the SDGs and compared this with the author’s qualitative responses.*

 2) As the authors note there is a disconnect between authors reporting that they are working (directly or indirectly) on societal relevant topics vs. the “impact” (that is, citations) that they are seeking in their work. Further exploration is needed on whether the respondents misinterpreted the first question or if it was worded so vaguely (“indirectly”) as to be meaningless. Note that much “basic” research in the Earth, environmental, and space science has widespread indirect impacts.

 Much real time “basic-science” data about the Earth is used in the GPS system, weather predictions, or other uses, e.g. For examples, see this discussion here that I was involved with:

 https://eos.org/editors-vox/earth-and-space-science-for-the-benefit-of-humanity and the linked papers. Indeed many grant applications require a statement regarding impacts. Similarly, results for the question on impact expected by the authors are used in comparison. I also wonder if the wording and reality of the scope of published papers drove this response (that is, many have an indirect vs. direct impact) and the response was viewed as a direct impact.

*A comment has been added on this in the revised submission to note further research would be useful to better understand the motivations and responses.*

 3) The authors list a number of actions T&F are taking or should take. Interestingly, T&F has not signed DORA—as Springer-Nature and Elsevier have now signed (whatever one thinks of that), Wiley and T&F are the major publishers who have not (many individual society journals published with Wiley have). Perhaps the authors could indicate why or why not that would be appropriate and how to leverage that impact. Here’s a recent editorial from a T&F publication: https://www.tandfonline.com/doi/full/10.1080/10919392.2018.1522774

*Pleasingly, Taylor & Francis has since signed DORA, but no comment has been made in the article, as the policy-related points have been removed.*

 4) The authors indicate what some stakeholders, especially publishers, might do. In the Earth environment and space sciences, there are several leading global societies. These are not mentioned. What is their role? Many have missions aligned with providing benefits to society and many are involved in science communication, policy, outreach and training/mentoring (moreso than most commercial publishers and indeed universities). Indeed this might be an argument to focus on publishing with a society versus a commercial title, where these resources are more directly leveraged.

*This was an oversight from the previous submission. As we have removed the policy discussion, we haven’t elaborated on this further, but we acknowledge the mission focus of many of the leading societies and their importance in shaping the behaviours of researchers in their communities.*

**Other items:**

 The authors argue that it is surprising that JIF is important to researchers but that they don’t always/regularly choose the highest JIF journals when submitting. This is because researchers know rejection rates and do optimization around likelihood of success (or they don’t want to waste their time, which is also important).

*We agree that likelihood of success is one of the main drivers in the decision-making of authors when selecting a journal and included a paragraph on whether the article was finally published in the first/second/third-or-more choice. We also suggest that speed of publication/time to first decision, and the journal’s relevance to the community are other important drivers in addition to acceptance rate and Impact Factor.*
